# Rigidity Emerges during Antibody Evolution in Three Distinct Antibody Systems: Evidence from QSFR Analysis of Fab Fragments

**DOI:** 10.1371/journal.pcbi.1004327

**Published:** 2015-07-01

**Authors:** Tong Li, Malgorzata B. Tracka, Shahid Uddin, Jose Casas-Finet, Donald J. Jacobs, Dennis R. Livesay

**Affiliations:** 1 Department of Bioinformatics and Genomics, University of North Carolina at Charlotte, Charlotte, North Carolina, United States of America; 2 Department of Formulation Sciences, MedImmune Ltd., Cambridge, United Kingdom; 3 Analytical Biochemistry Department, MedImmune LLC, Gaithersburg, Maryland, United States of America; 4 Department of Physics and Optical Science, University of North Carolina at Charlotte, Charlotte, North Carolina, United States of America; Max Planck Institute for Biophysical Chemistry, GERMANY

## Abstract

The effects of somatic mutations that transform polyspecific germline (GL) antibodies to affinity mature (AM) antibodies with monospecificity are compared among three GL-AM Fab pairs. In particular, changes in conformational flexibility are assessed using a Distance Constraint Model (DCM). We have previously established that the DCM can be robustly applied across a series of antibody fragments (VL to Fab), and subsequently, the DCM was combined with molecular dynamics (MD) simulations to similarly characterize five thermostabilizing scFv mutants. The DCM is an ensemble based statistical mechanical approach that accounts for enthalpy/entropy compensation due to network rigidity, which has been quite successful in elucidating conformational flexibility and Quantitative Stability/Flexibility Relationships (QSFR) in proteins. Applied to three disparate antibody systems changes in QSFR quantities indicate that the VH domain is typically rigidified, whereas the VL domain and CDR L2 loop become more flexible during affinity maturation. The increase in CDR H3 loop rigidity is consistent with other studies in the literature. The redistribution of conformational flexibility is largely controlled by nonspecific changes in the H-bond network, although certain Arg to Asp salt bridges create highly localized rigidity increases. Taken together, these results reveal an intricate flexibility/rigidity response that accompanies affinity maturation.

## Author Summary

Antibodies are protective proteins used by the immune system to recognize and neutralize foreign objects through interactions with a specific part of the target, called an antigen. Antibody structures are Y-shaped, contain multiple protein chains, and include two antigen-binding sites. The binding sites are located at the end of the Fab fragments, which are the upward facing arms of the Y-structure. The Fab fragments maintain binding affinity by themselves, and are thus often used as surrogates to student antibody-antigen interactions. High affinity antibodies are produced during the course of an immune response by successive mutations to germline gene-encoded antibodies. Germline antibodies are more likely to be polyspecific, whereas the affinity maturation process yields monoclonal antibodies that bind specifically to the target antigen. In this work, we use a computational Distance Constraint Model to characterize how mechanical properties change as three disparate germline antibodies are converted to affinity mature. Our results reveal a rich set of mechanical responses throughout the Fab structure. Nevertheless, increased rigidity in the VH domain is consistently observed, which is consistent with the transition from polyspecificity to monospecificity. That is, flexible antibody structures are able to recognize multiple antigens, while increased affinity and specificity is achieved—in part—by structural rigidification.

## Introduction

The variable region of an antibody is composed of a structurally conserved fold that contains six complementarity-determining regions (CDRs), also known as hypervariable regions. The six CDRs, three on the light chain (L1, L2 and L3) and three on the heavy chain (H1, H2 and H3), are known to be responsible for the majority of antibody-binding interactions. Antibody evolution starts with the assembly of germline (GL) antibodies in B and T cell progenitors through the recombination of V, D, and J gene segments [1]. Theoretically, V-(D)-J recombination could generate 2.3 × 10^12^ antibody variable domains [2], which is far less than the number of epitopes on foreign antigens to which one could be exposed. Therefore, the GL antibodies undergo further cycles of somatic mutations for affinity maturation (AM) and specificity improvement as the immune response proceeds, which can produce an astronomical number of unique antibodies.

A variety of biochemical and structural studies reveal that the same germline gene-encoded antibodies allow promiscuous binding to diverse antigens, and even the same antigens by quite different somatic mutations [3–5]. Structural diversity in the antigen-binding site accounts for the immense breadth of binding of the antibody repertoire. Two hypotheses, conformational flexibility and the induced-fit models, are commonly invoked to explain the conformational changes of antibodies during affinity maturation. Conformational flexibility assumes GL antibodies retain a degree of structural plasticity in their backbone in order to bind a number of different unrelated antigens, a capacity referred to here as polyspecificity [3, 6]. In contrast, the induced-fit model supposes that conformational changes are induced as antigens binding to the Ab [7–9]. Regardless of the explanation, it is clear that flexibility/rigidity is changed, which is closely related to the binding affinity and specificity of antigens [4, 5, 10–13].

There is much evidence to suggest that mature antibodies, especially within the CDRs, are inherently more rigid than their GL precursors. Lipovsek et al. [14] demonstrated that constricting the flexibility of CDRs with inter-loop disulfide bonds enhanced the affinity of immunoglobulin interactions. Schmidt et al. [15] studied a broadly neutralizing influenza virus antibody using long-scale molecular dynamics and demonstrated that maturation rigidifies the initially flexible heavy-chain CDRs, which accounts for most of the affinity gain. Jorg et al. [16] applied three-pulse photon echo spectroscopy and molecular dynamic to explore the flexibility of mature 4-4-20 antibody and found that the binding site of the mature antibody is significantly rigidified compared to that of the GL, and that the increased rigidity occurs via increased coupling within and between CDR loops and the antibody framework. Finally, Manivel et al. [17] proposed that more unfavorable entropy changes are associated with ligand binding within GL antibodies compared to AM.

Although genetic and biochemical studies have revealed the nature and origin of the sequence diversity of antibodies, the mechanisms by which the somatic mutations change the flexibility of the antibody-binding site is not well understood. Accurate assessment of the flexibility of the CDRs might be particularly important to further understand the thermodynamics of immunoglobulin binding. Flexibility of the CDRs is related to the polyspecificity by providing the capacity of a single binding site to bind different ligands. Molecular dynamics (MD) is commonly used to quantify protein “flexibility” at a very detailed level. However, quantifying protein motions characterized by MD trajectories using standard metrics such as root mean squared fluctuations (RMSF) or assessing essential dynamics by principal component analysis capture atomic motions with large amplitudes. While the phrase “flexibility” is often used interchangeably with mobility, there is a technical difference. For example, an α-helix constitutes a rigid substructure, yet it can simultaneously be highly mobile if its position as a rigid body undergoes large fluctuations. Conversely, a flexible region may serve as a hinge point to facilitate relative motions, but the hinge itself need not be mobile. Although flexibility and mobility are distinct properties, this distinction is typically not made in the literature.

Flexibility is characterized by network rigidity as a direct mechanical property of molecular structure. The Distance Constraint Model (DCM) characterizes protein flexibility in a thermodynamically appropriate way [18, 19]. The DCM has been successfully applied for many protein systems such as RNase H [20], periplasmic binding proteins [21], thioredoxin [22], lysozyme [23, 24], and **β**-lactamase [25]. Collectively, these results reveal that conformational flexibility is very sensitive to perturbation (e.g., mutation and ligand binding). Moreover, these flexibility changes frequently propagate over long distances. Recently, we characterized the effects of mutation to single chain Fv (scFv) fragments of the anti-lymphotoxin-**β** receptor antibody using the DCM [26]. Statistically significant changes in the distribution of both rigidity and flexibility within the molecular structure is typically observed, where the local perturbations often lead to distal shifts in flexibility and rigidity profiles.

In this report, we similarly characterize the effects of somatic mutations on the flexibility/rigidity changes by analyzing three GL-AM antigen-binding fragment (Fab) pairs. Interestingly, CDR H3 loop is rigidified after affinity maturation in all three cases. We observe a rich mixture of increased rigidity and flexibility along the backbone, and many of these changes are significantly long-ranged. In many instances specific hydrogen bonds or salt bridges that form in regions where there is tight side chain packing play an important role in rigidifying CDRs during the maturation process. The accompanying loss of conformational entropy due to this increase in rigidity near the mutation site is an enthalpy-entropy compensation mechanism that the DCM captures well through network rigidity. In addition, molecular couplings that describe flexibility and rigidity correlations between residues are frequently enhanced by somatic mutations. The structural plasticity of GL antibodies and associated trends in how rigidity and flexibility profiles redistribute upon maturation likely represent general mechanisms used by the immune response and could be used to guide design high affinity and selective antibodies for desired function.

## Methods

### The Distance Constraint Model

The DCM is defined in terms of an all-atom free energy decomposition (FED) scheme combined with constraint theory. Atomic structure is mapped onto a graph where vertices represent atomic positions and edges describe intramolecular interactions that fix the distance between atomic positions. This graph defines a mechanical framework that is characterized by its constraint topology. A Pebble Game (PG) algorithm identifies all rigid and flexible regions [27, 28], which can provide statistically significant explanations of intramolecular couplings [29]. An ensemble of graphs is considered to account for fluctuations in constraint topologies due to the breaking and forming of H-bonds and packing interactions. The DCM generates a Gibbs ensemble of graphs, where each graph is weighted by a Boltzmann factor given by *xp*(−*βG*). The free energy of a graph is calculated from a FED where each constraint is associated with a component enthalpy and entropy. The total enthalpy of a graph is the sum over all enthalpy contributions. However, as described below, the total entropy accounts for nonadditivity [23, 30, 31] due to network rigidity. Within the minimal DCM [18, 32] the number of native-like torsion constraints, *N*_*nat*_, and number of H-bond constraints, *N*_*hb*_ specify a macrostate. Native torsion states have lower energies and entropies relative to disordered torsion states, meaning they correspond to good packing interactions. As a result, protein stability is described in terms of both intramolecular packing and the H-bond network. Note that salt bridges are considered to be a special case of H-bonds.

The two order parameters, (*N*_*hb*_, *N*_*nat*_), define a macrostate of a protein in terms of its constraint topology, from which a free energy functional is constructed as:

$$G\left(N_{hb},N_{nat}\right)=U\left(N_{hb}\right)-u_{sol}N_{hb}+v_{nat}N_{nat}-T\left[S_{conf}\left(N_{hb},N_{nat}|\delta_{nat}\right)+S_{mix}\left(N_{hb},N_{nat}\right)\right]$$ (1)

where *U* is the intramolecular H-bond energy, *u*_*sol*_ is an average H-bond energy to solvent that occurs when an intramolecular H-bond breaks, *v*_*nat*_ is the energy associated with a native-like torsion, *S*_*conf*_(*N*_*hb*_, *N*_*nat*_) is the conformational entropy and *S*_*mix*_(*N*_*hb*_, *N*_*nat*_) is the mixing entropy of the macrostate associated with the number of ways of distributing *N*_*nat*_ native-torsions and *N*_*hb*_ H-bonds within the constraint topology. Three phenomenological parameters, {*u*_*sol*_, *v*_*nat*_, *δ*_*nat*_}, effectively account for overall structural shape and solvent interactions. Conformational entropy, *S*_*conf*_, is calculated over the set of independent constraints identified by the PG using:

$$S_{conf}\left(N_{hb},N_{nat}\right)=R{\langle \sum_{t\in hb}q_{t}\gamma_{t}+Q_{nat}\delta_{nat}+Q_{dis}\delta_{dis}\rangle }_{graphs}$$ (2)

where the index *t* spans over all H-bond constraints in the input structure, and each H-bond has a *q*_*t*_ of either {0, 1, 2, 3, 4 or 5} to count the number of distance constraints that are independent based on the PG. Note that in the mDCM, each H-bond is modeled using five distance constraints. Hence, it is possible that all five constraints are independent (i.e. *q*_*t*_ = 5) or all five constraints are redundant (i.e. *q*_*t*_ = 0) or any range in between if the *t-*th H-bond is present, and *q*_*t*_ = 0 if the *t*-th H-bond is not present (i.e. broken). For each independent H-bond distance constraint, *R*_*γt*_ is the conformational entropy contribution. The details of these calculations depend greatly on the number of H-bonds present in the protein and where they are distributed. Moreover, there is a strong dependence on the number and location of torsion constraints within the protein. The macrostate stratifies the total number of H-bonds and total number of native torsions.

All native-like and disordered torsion constraints respectively contribute *R* *δ*_*nat*_ and *R* *δ*_*dis*_ to the conformational entropy when they are independent. Taking advantage of the degeneracy, the variable *Q*_*nat*_ is the total number of native-like torsions that are independent and *Q*_*dis*_ is the total number of disordered torsions that are independent. The various *q*_*t*_, *Q*_*nat*_ and *Q*_*dis*_ values in Eq (2) are calculated for each mechanical framework (graph) using the PG, and the conformational entropy is obtained as an ensemble average over many graphs as denoted by the averaging brackets [18]. Monte Carlo sampling is used to sample networks at each macrostate value (*N*_*hb*_, *N*_*nat*_). Typically, 200 samples per macrostate provide enough sampling to obtain sufficiently accurate statistics.

Lastly, there is a critically important step that must be executed when determining if a constraint is independent or redundant. When the PG is used to calculate whether a constraint is independent or redundant during a recursive process of building the PG graph one constraint at a time [28], the constraints are placed in preferential order from lowest to highest component entropies. With this preferential ordering, the calculation of conformational entropy provides a lowest possible upper bound estimate. Conceptually, total conformational entropy reflects the minimal set of the most constrictive yet independent interactions. Solvation free energy contributions are modeled by the phenomenological *u*_*sol*_ and *v*_*nat*_ parameters [33] that are conjugate to the intramolecular H-bonds and packing order parameters respectively. While mutations are known to quantitatively affect solvation free energies [34], the same *u*_*sol*_ and *v*_*nat*_ parameters are used throughout because the changes are not expected to be large here due to the overall structural similarity across the dataset.

### Dataset

We created a dataset of three pairs of GL-AM antibody Fabs that include anti-fluorescein (FA), anti-CD3 T-cell receptor (CA) and esterase catalytic (EA) antibodies (Table 1). In this dataset, except for the GL Fabs of FA and CA, all the X-ray structures are available. The structures of GL Fabs of both FA and CA were modeled using their corresponding AM structures as templates by SCWRL4 [35]. The melting temperatures (*T*_*m*_) of FA(AM), CA(GL) and CA(AM) are available. S1 Table summarized the potential germline genes of the three antibodies based on the bioinformatics analysis of the closet human germline sequences by the alignments of the sequences of maturated antibodies with those from the germline sequence database. Across the three antibodies, only one or two segments may come from the same genes and other segments are from different GL genes, meaning the three example systems do not belong to the same GL family and suggesting wide applicability of our results. S2 Table shows the experimentally measured binding affinities of the three antibodies in the dataset with their antigens. The affinity increases from GL to AM is at least 32-fold in FA and 7.3 fold in EA. The GL CA antibody does not bind antigen, whereas the affinity for the AM antibody is Kd = 0.64 μM. The number and identity of mutations between AM and GL are summarized in S3 Table.

**Table 1:** Dataset of the GL-AM pairs for rigidity/flexibility analysis.

Antibody Fab	GL/AM	PDB ID	Residue	*T*_*m*_ (K)	Total # of clusters	# conformations in 10 largest clusters^b^
Anti-Fluorescein	GL	NA^a^	437	NA	12	1987
	AM	1FLR	437	326 [47]	13	1839
Anti-CD3	GL	NA^a^	432	343 [71]	13	1858
	AM	1SY6	432	344 [71]	14	1909
Esterolytic catalytic Ab	GL	1AJ7	431	NA	11	1986
	AM	1GAF	431	NA	13	1930

a. The structures were modeled based on their corresponding AM antibody structure.

b. Each clustering was performed for 2,000 conformations extracted from 100 ns molecular dynamics trajectory.

### Molecular Dynamics Sampling

We employed molecular dynamics (MD) simulations to generate an ensemble of conformations (10 representative structures) for subsequent DCM analysis. The advantage of using multiple structures instead of a single structure is that sensitivity to structural artifacts is diminished and uncertainties can be estimated [26]. Each structure in the dataset was simulated for 100 ns using Gromacs 4.5.5 [36, 37] in the NVT ensemble with the AMBER99SB-ILDN force field [38]. The structures were solvated by adding 10.0 Å of TIP3P water [39] in a cubic box (counter ions are also added to neutralize charge). Before production, the systems were minimized for 5,000 iterations in Gromacs, followed by 1 ns of NPT and 1 ns of NVT equilibration. Pressure (1 atm) was regulated using the extended ensemble Parrinello-Rahman approach [40] and temperature (300 K) was controlled by a Nose-Hoover temperature coupling [40, 41]. A nonbonded cutoff of 10.0 Å was used, and Particle-Mesh-Ewald [42] accounts for long-range electrostatic interactions. All bonds to hydrogen atoms in proteins were constrained using LINCS [43], whereas bonds and angles of water molecules are constrained by SETTLE [44], allowing for a time step of 0.002 ps.

### DCM Parameterization

The phenomenological parameters, {*u*_*sol*_, *v*_*nat*_, *δ*_*nat*_}, are ideally obtained by fitting to experimental heat capacity curves from DSC. In our two recent reports [26, 45], we established parameter ranges for various antibody fragment sizes and the parameters are relatively conserved within the same antibody fragment. In addition, we showed that the mDCM could be parameterized in the absence of experimental heat capacity curves assuming the melting temperature is known or estimated. Therefore, because of the lack of experimental C_*p*_ curves for our dataset we obtained the parameters for the six antibodies by two ways. If the *T*_*m*_ value of the antibody is known, we fit the structure to a presumed similar experimental *C*_*p*_ curve from a commercial anti-VEGF antibody Fab, Bevacizumab, which has one peak corresponding to the *T*_*m*_ at 347K [46]. Prior to the fitting, the *T*_*m*_ of the target-experimental *C*_*p*_ curve was shifted to the true experimental *T*_*m*_ (326K) [47] For the antibodies without experimental *T*_*m*,_ we used the same parameters from FA (S4 Table).

### Quantitative Stability/Flexibility Relationships

In addition to calculating thermodynamic properties, the mDCM calculates a number of mechanical properties that are ensemble averaged. Taken together, the mDCM produces Quantitative Stability/Flexibility Relationships (QSFR) of the protein. For example, large extended rigid sub-structures, punctuated by flexible loops, are prevalent at low temperatures, whereas the protein is primarily flexible in the denatured ensemble at temperatures greater than the *T*_*m*_ defined by the heat capacity peak. The backbone Flexibility Index (FI) and the Cooperativity Correlation (CC) serve as useful QSFR metrics for characterizing mechanical properties within a protein [48].

The FI is an ensemble average over the quantity *f*_*i*_ = (*h*_*i*_ *−l*_*i*_) that is calculated for each constraint topology as follows. When the *i*-th rotatable bond can rotate within a flexible region, the number of rotatable bonds that can rotate (distinct hinge motions) within that flexible region is counted, and denoted as H. The number of independent disordered torsions within that flexible region is also counted, and denoted as A. The value *h*_*i*_ = A/H represents the density of independent degree of freedom (DOF) within that flexible region, and it is assigned to all H rotatable bonds within. Conversely, if the *i*-th rotatable bond is locked within an over-constrained region, the total number of rotatable bonds that are locked are counted and denoted as L. The number of redundant constraints within that over-constrained region is also counted, and denoted as B. The value *l*_*i*_ = B/L represents the density of redundant constraints within that over-constrained region, and it is assigned to all L locked bonds within. In the special case that B = 0, the locked bond is called isostatic, but this distinction is lost in FI due to ensemble averaging.

The CC matrix is calculated similarly to FI; however, mechanical couplings are being tracked. That is, for a given constraint topology, the decomposition of regions as described above also yield which pair of rotatable bonds are in the same flexible region or same rigid region. If the *i*-th and *j*-th rotatable bond are in the same flexible region, the matrix element CC_*ij*_ = *h*_*i*_ (recall *h*_*i*_ = *h*_*j*_). If they are in the same rigid region, the matrix element CC_*ij*_ = –*l*_*i*_ (recall *l*_*i*_ = *l*_*j*_). If the pair of rotatable bonds are not within the same distinct region, the matrix element CC_*ij*_ = 0 and this pair of rotatable bonds are not correlated. The size of the CC matrix representing the backbone is nominally 2N·2N because the phi and psi torsions are tracked along the backbone. However, generally the CC matrix is slightly smaller in size because proline has only the psi rotatable bond. Although the backbone rotatable bonds within a residue can be averaged to arrive at a N·N matrix, the CC matrix that we typically use, as is the case here, show all rotatable angles.

There are multiple ways to ensemble average mechanical properties and other physical observables. Note that because Boltzmann factors weight macrostates differently within the free energy landscape, the most probable constraint networks depend on temperature. In this report, we average over all macrostates corresponding to the native basin to focus on equilibrium fluctuations in the folded protein at *T = T*_*m*_. As such, both the FI metric and the CC matrix represent the average native state characteristics at *T = T*_*m*_.

### Comparative Analyses

In this report, residue numbering is based on the Kabat scheme [49]. The QSFR properties are calculated for each representative structure, and a second average over 10 representative structures is performed with a weighting that is based on cluster size. To compare mutant QSFR properties to the wild type, we use a Z-score (Eq 3) to discern differences between the GL and AM results across the 10 representative structures.

Z=(x¯mut−x¯wt)σmut210+σwt210 (3)

The value of 10 corresponds to the number of representative structures considered. Cluster weightings are included when calculating the averages and standard deviations of a quantity over the 10 representative structures. Using a conservative Z-score cut-off, statistically significant changes are deemed to occur when |Z-scores| are greater than 2.33, corresponding to a p-value of 0.01. Further, large changes are deemed to occur when |Z-scores| are greater than 3.33, which corresponds to a p-value of 0.0005. That is, the odds of a moderate change occurring by random chance are 1 in 100, and the odds of a large change occurring by random chance are less than 1 in 2300. No change is assigned when Z-scores are between ±2.33.

## Results and Discussion

### Analysis of the Dataset

**Fig 1:**
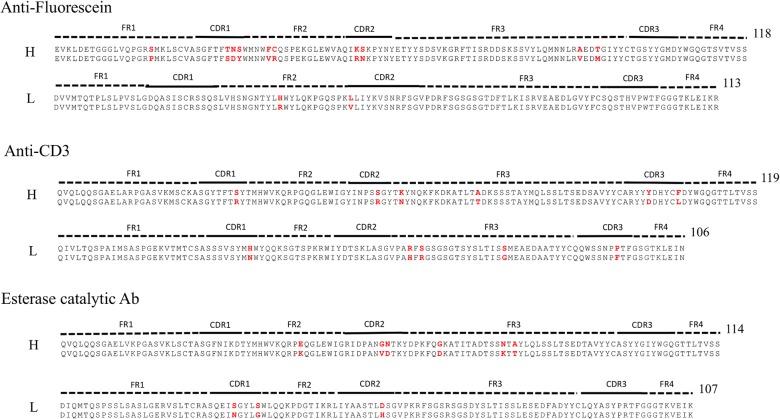
Sequences of the three considered Fab fragments. Sequence alignments comparing the three GL/AM pairs for the anti-fluorescein, anti-CD3 and esterase catalytic antibodies. FRs, CDRs regions and the lengths of sequences are indicated on top of each alignment. Yellow shading shows introduced amino acid mutations.

**Fig 2:**
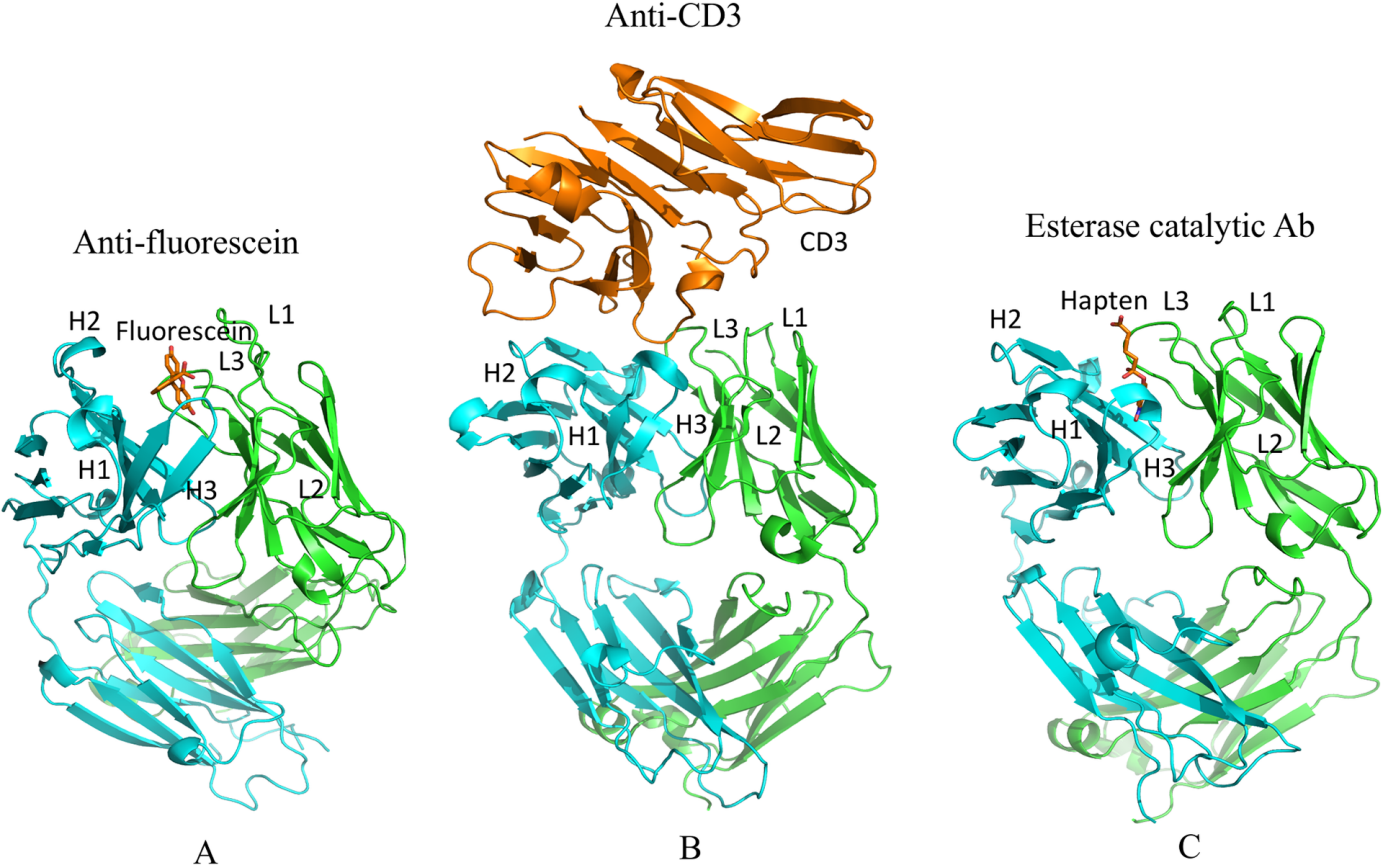
Structures of the three Fab fragments bound with antigens (fluorescein, CD3 and hapten, respectively) are shown. H and L chains are colored in cyan and green, respectively; epitope is colored in orange.

Three GL-AM antibody pairs including anti-fluorescein, anti-CD3 T-cell receptor and esterase catalytic antibodies were compiled for rigidity/flexibility analysis. For each pair we compare the AM sequence to the GL sequence via sequence alignments (Fig 1). The numbers of mutations located in the Fab and CDRs with respect to the GL sequence are 12/6, 9/6 and 9/5 for FA, CA and EA, respectively, indicating that 50% or more of the mutations occur in CDRs. Fig 2 shows the binding modes of the antigens in their AM antibodies. It is noted that antigen fluorescein and hapten 5-(para-nitrophenyl phosphonate)-pentanoic acid are located in similar sub-regions of the binding site, which facilitate them to interact directly with the CDR-H3 and CDR-L3 loops. Similarly, one epitope loop of antigen CD3 inserts into the same sub-regions, while others make contact with CDR-H1 and CDR-H2 loops. S1 Fig summaries the changes of amino acid propensity during affinity maturation. An apparent trend is that charged residues are favorable in the mature sequences while polar residues are unfavorable (S5 Table).

### Generation of Representative Structures by Molecular Dynamics

**Fig 3:**
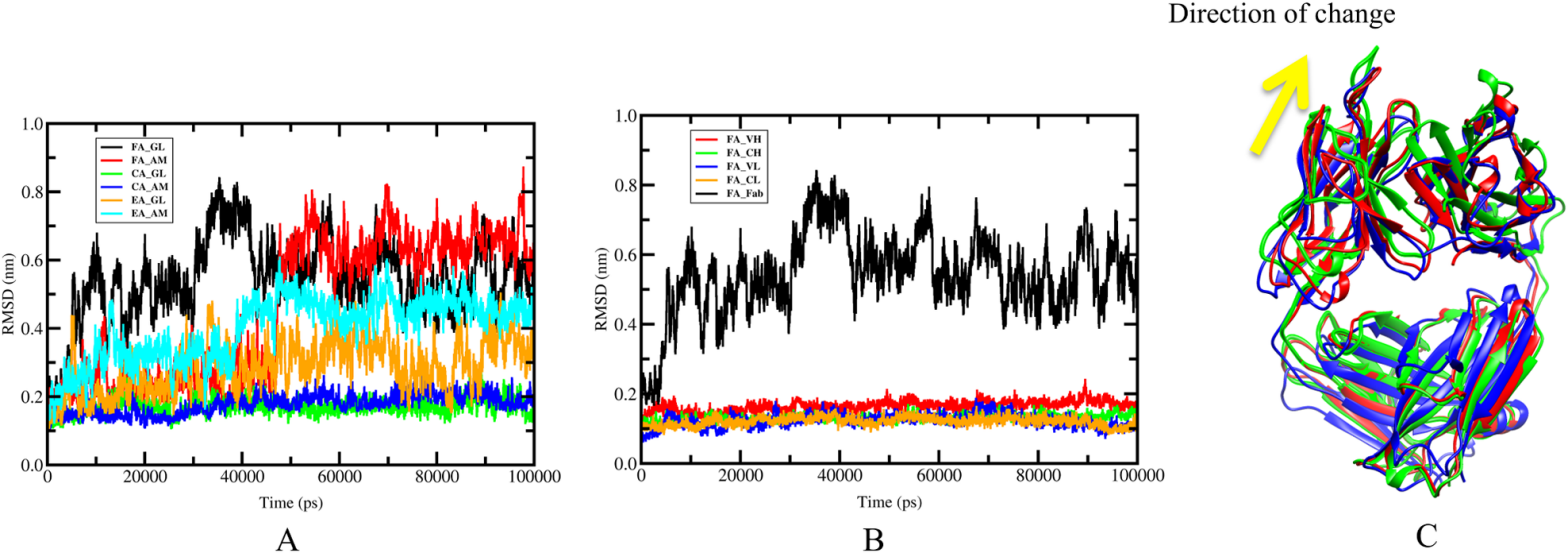
Molecular dynamics trajectories. FA, CA and EA represent anti-fluorescein, Anti-CD3 and esterase catalytic antibodies, respectively. (A) Root mean square deviations (RMSDs) of Cα are provided each of the molecular dynamics trajectories. The FA(GL) and FA(AM) exhibit larger RMSDs than other antibodies due to change of domain-domain reorientation. (B) Global RMSDs of for the full FA(GL) Fab and individual RMSDs for each domain. All the four domains (VH, VL, CH and CL) show much lower RMSDs than global RMSDs. The small fluctuations within the domains highlight that the global fluctuations are caused by slippage along the domain interface, where the four domains are continually rearranging relative to each other. (C) The slippage along the domain interfaces is indicated in panel (B), where different colors represent snapshots occurring at: 20 ns (red), 40 ns (blue), and 80 ns (green).

Our previous study of the stability and flexibility of wild-type and stable mutants of scFv using mDCM indicate that an average over the most weighted ten representative structures sampling by MD reduces the statistical variance in mean QSFR properties to a point that is less than the level of accuracy that can be expected from the employed phenomenological mDCM underlying the calculations [26]. In addition, the modeled structures require further conformational refinement. Thus, we performed MD on all six Fab structures to generate a set of representative conformations for mDCM analysis. Root mean square distances (RMSD) of Cα atoms are plotted in Fig 3. In all six cases the fluctuations within the constituent Fv and Fc regions from the Fab are well converged across the 100 ns trajectory (S2 Fig). The same is true in each of the individual Ig-folds. While the overall RMSD values approach 8 Å in the GL and AM trajectories, these large fluctuations are simply due to reorientations between the Fv and Fc regions (cf. Fig 3C). Due to the immense flexibility within the linker regions, these rearrangements are continuously present in the native ensemble as equilibrium fluctuations, and these fluctuations have been noted previously by both simulation and experiment [50, 51]. Therefore, these particularly large fluctuations do not indicate poor convergence, and no added benefit would be achieved by simply simulating the systems longer. Moreover, it is worth noting the pronounced Fv/Fc rearrangements occurring in both the GL and AM trajectories eliminates the mutant modeling process as the cause of these fluctuations. It is interesting to note that the FA, one of two anti-hapten systems, has the largest domain-domain fluctuations; antibodies raised to haptens tend to be more susceptible changes in conformation upon binding than anti-protein antibodies [52]. On the other hand, the catalytic EA antibody has the smallest global RMSD fluctuations, so it is impossible to conclude anything with respect to the conformational fluctuations based on this hapten vs. protein antigen distinction.

A total of 2,000 evenly spaced frames from each trajectory were clustered using the KCLUST module [53] from the MMTSB tool set [54] based on the RMSD of all heavy atoms. Table 1 summarizes the number of conformations represented by each cluster. We adjust the cluster radii to maintain around 20 total clusters, where the ten largest represent 92 to 99% of the total conformations. A representative structure is identified as the centroid from each of the ten largest clusters, which are then subsequently energy minimized and used as input to the mDCM. A weighted average of all mDCM properties is taken over the ten representative structures, where the total number of structures within the cluster containing a given representative structure defines its weight. After MD simulation and clustering, H++ [55] is employed to account for protonation state fluctuations by calculating ionization properties by considering residue p*K*_*a*_ values followed by a final minimization [56].

### Rigidity Changes during Affinity Maturation

**Fig 4:**
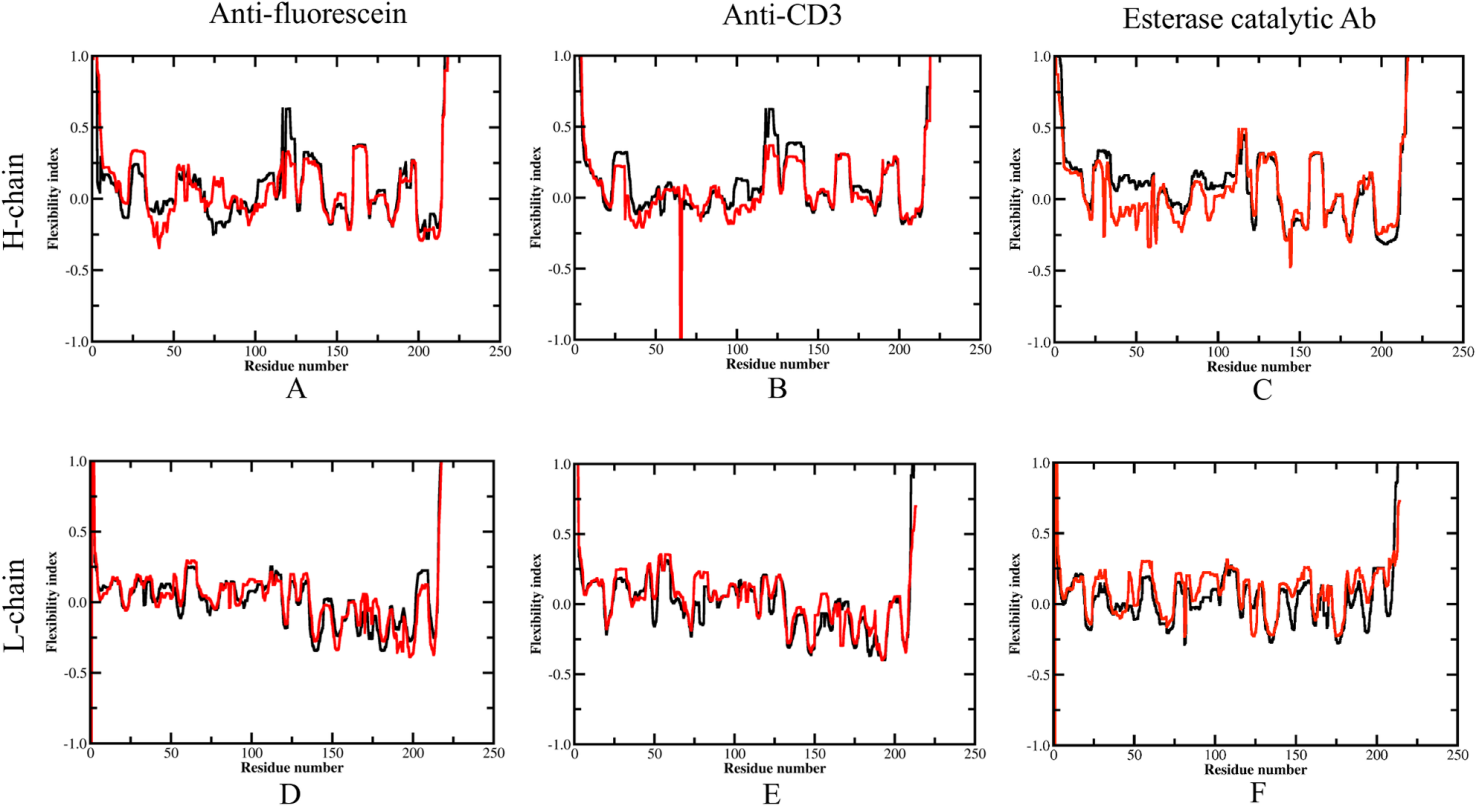
Average flexibility index for each antibody H and L chains. (A) and (D) are flexibility index for anti-fluorescein antibody H and L chain, respectively. (B) and (E) for anti-CD3 antibody. (C) and (F) for esterase catalytic antibody. Black and red curves represent flexibility index for GL and AM antibodies, respectively. Reported values correspond to the appropriate weighted average (defined in methods) over 10 representative structures sampled from the MD trajectory.

Backbone flexibility as described by the flexibility index (FI) for each antibody within the dataset is shown in Fig 4. Positive FI values indicate flexibility, whereas negative values indicate rigidity. Across the alignment, most secondary structure elements are determined to be rigid, whereas intervening loops are flexible. The termini and linker regions (residue 112–120) between the VH and CH domains shows considerable flexibility, which is in agreement with the large fluctuations within domains observed by molecular dynamics (Fig 3). Despite this overall qualitative similarity, there are quantitative differences throughout.

To assign statistical significance to the observed changes, we recast the differences between the GL and AM antibodies as Z-scores. The Z-scores for each GL-AM pairs are plotted against residue number in S3 Fig where all rigidity/flexibility differences are classified as no significant change (|Z-score| < 2.33), moderate change (2.33 < |Z-score| < 3.33) and large change (|Z-score| > 3.33). Table 2 counts the number of residues with altered rigidity/flexibility. Across the Fabs and CDRs, the overall number of residues with increased rigidity (both are 52%) is slightly higher than increased flexibility (both are 48%). These percentages indicate that both the Fab and antibody-binding site, as a whole, maintains a global balance between rigidity and flexibility during affinity maturation. Therefore, Le Châtelier’s principle, stating that an equilibrium shift will occur to offset the perturbation and a new equilibrium is established, can be applied as a rule of thumb to make credible predictions of mutation effects on protein flexibility. That is the effects of affinity-improved mutations on the rigidity⇔flexibility equilibrium within the native state ensemble manifest themselves through enthalpy-entropy compensation as the protein structure adjusts to restore the global balance between the two. It is also interesting to highlight that increased rigidity in CDR-H3 is observed in all three AM antibodies.

**Table 2:** Frequency of increased rigidity vs. increased flexibility.

Antibody	Chain	Flexibility increases^a^		Rigidity increases^a^	
		2.3 ≤ *x *< 3.3	3.3 ≤ *x*	-3.3 < *x *≤ -2.3	*x ≤ *-3.3
		*whole FABs*			
Anti-Fluorescein	H	6	19	19	3
	L	26	15	23	1
Anti-CD3	H	2	0	20	29
	L	16	20	5	3
Esterase catalytic Ab	H	2	3	21	37
	L	29	38	3	1
Total		81	95	91	74
		*Complementarity Determining Regions*			
Anti-Fluorescein	H	0	8	5	0
	L	11	6	0	0
Anti-CD3	H	0	0	8	11
	L	1	3	0	0
Esterase catalytic Ab	H	0	0	15	10
	L	11	14	0	0
Total		23	31	28	21
		*CDR H3*			
Anti-Fluorescein		0	0	5	0
Anti-CD3		0	0	7	3
Esterase catalytic Ab		0	0	7	3
Total		0	0	19	6
		*CDR L2*			
Anti-Fluorescein		5	3	0	0
Anti-CD3		4	3	0	0
Esterase catalytic Ab		5	2	0	0
Total		14	8	0	0

^a^ The counts of amino acids belonging to this classification.

The increase in CDR-H3 rigidity is in good agreement with a backbone entropy study for immunoglobulin (CDRs) from the crystal structures of 34 low-affinity T-cell receptors and 40 high-affinity Fabs. Specifically, it has been demonstrated that loss of backbone entropy in CDR3 correlates significantly with the kinetic and affinity constants of the 74 selected complexes [11]. CDR-H3 likely plays a critical role in determining the evolution of antibodies because junctional amino acids introduced by imprecise joining in the combinatorial rearrangement of VH, DH, and JH genes provide increased diversity of CDR-H3 [57]. Interestingly, structural analysis (Fig 2) shows that the antigens in all three AM antibodies contact directly with the CDR-H3, which suggests that the lower entropic penalty upon binding due to increased rigidity of CDR-H3 is most likely related to the affinity maturation and specificity to a specific antigen. Similar results were presented by Manivel et al [17] who found that antibody maturation essentially reflects modulations in entropy-control of the association, but not dissociation, step of the binding [12]. Another consistent change in all the three cases is the increased flexibility of CDR-L2. From a structural viewpoint, the CDR-H3 loop is located in the center of the Ag-combining site facing CDR-L2, it is possible that this loop not only affects the flexibility but also controls the angle between the VH and VL domains. Zimmermann et al. [10] reported that the whole antibody-binding site is rigidified during affinity maturation while our study indicates that an increase in rigidity only occurs in the CDR-H3 loop, but CDR loops in the light chain tend to become more flexible during affinity maturation.

**Fig 5:**
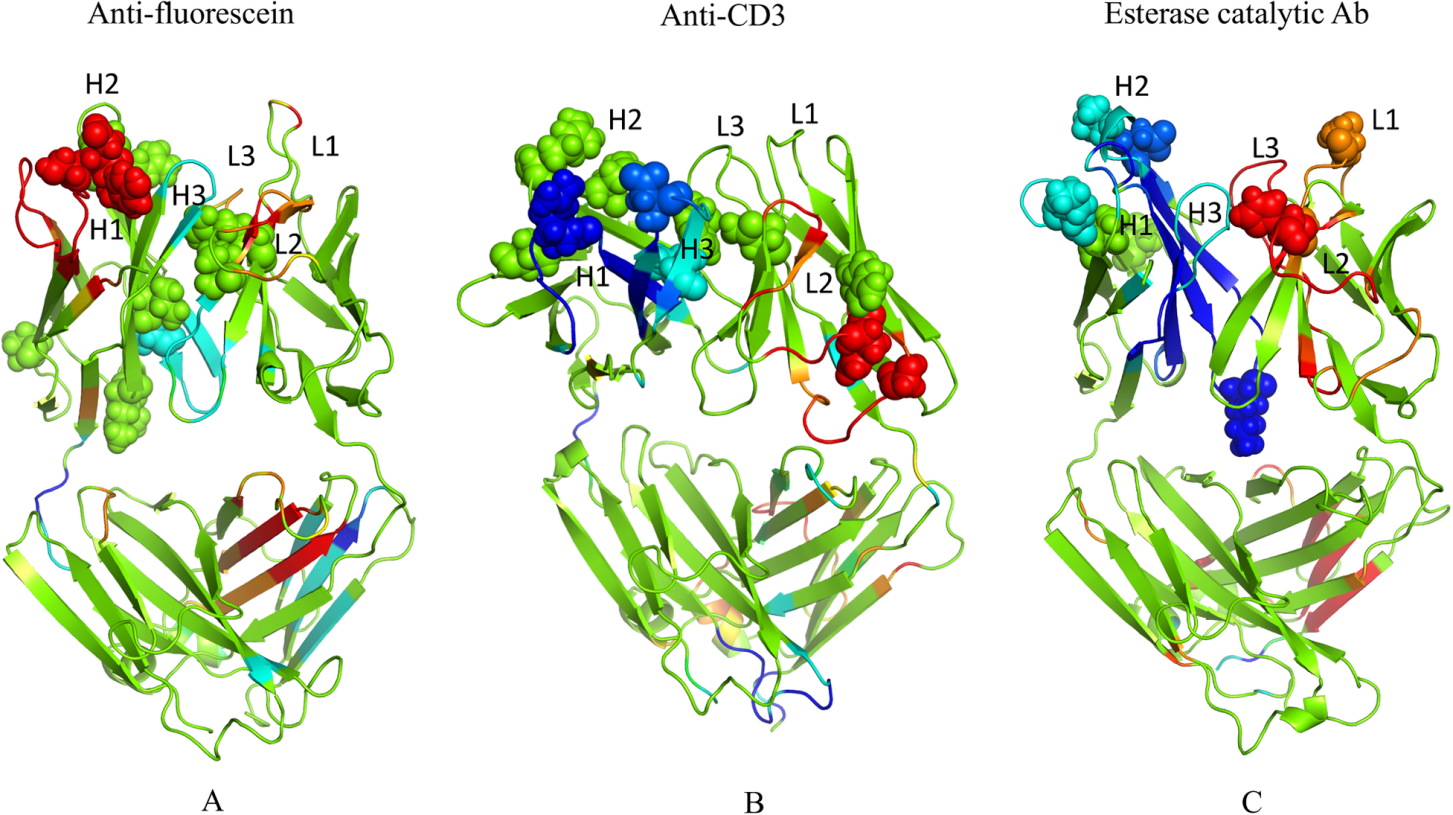
Changes in mechanical properties upon maturation. Differences in rigidity/flexibility between GL and AM antibodies across the dataset are projected onto the structures and indicated by color: green = no change; cyan and blue = moderate and large rigidity increases; and orange and red = moderate and large increases in flexibility. The mutations are displayed in spheres. In each case, the color represents a certain z-score range for differences that are defined within S3 Fig.

The Z-scores are mapped to structure using the same stratification of changes indicated above (cf. Fig 5). Note that changes tend to occur primarily in loop regions of the variable domains especially in CDRs. In FA, the CDR-H3 loop is moderately rigidified, whereas H1 and L2 both become flexible. In CA, both H1 and H3 loops become rigid, whereas L2 becomes flexible. In EA, all three heavy chain CDRs become rigid, whereas all the light chain CDRs become flexible. Taken together, our results show a wide array of flexibility and rigidity changes, but general trends of the VH domains becoming more rigid and the VL domains becoming more flexible is observed. It is also interesting to point out that while all of the mutations occur in the Fv fragment, there are changes in the Fc in all three cases. The mix of increased rigidity and flexibility occurring in the CL domain of FA is particular noteworthy (cf. Fig 5A).

### Mechanistic Details

Since the H-bond network (HBN) is a critical component to protein rigidity, we characterize the changes that occur in the HBN in response to somatic mutation to better understand the observed rigidity differences. We track H-bonds across the MD trajectories by comparing densities between GL and AM in each pair. The propensity of a H-bond to form between a specific potential donor-acceptor pair is measured by the fraction of occurrence of this H-bond over the 2,000 frames. For example, if a H-bond occurs in all 2,000 frames, the propensity of the H-bond is 1, whereas the value is 0.5 is assigned if it only occurs in half of the frames. The propensity for a residue to be involved in H-bond formation is defined as the sum of all H-bond propensities formed by its atoms. The HBNs for the GL and AM antibodies are provided in S4 Fig. The HBN differences are greater outside of secondary structures, while secondary structure H-bonds are more similar. Not surprisingly, this suggests that the preservation of secondary structure H-bonds are largely responsible for backbone flexibility to be well conserved, and why FI aligns well with secondary structure elements. Conversely, the largest H-bond differences involving side chains elucidate significant differences in rigidity properties. That is, a change in a handful of critically placed side chain H-bonds can drastically alter mechanical linkage properties.

**Fig 6:**
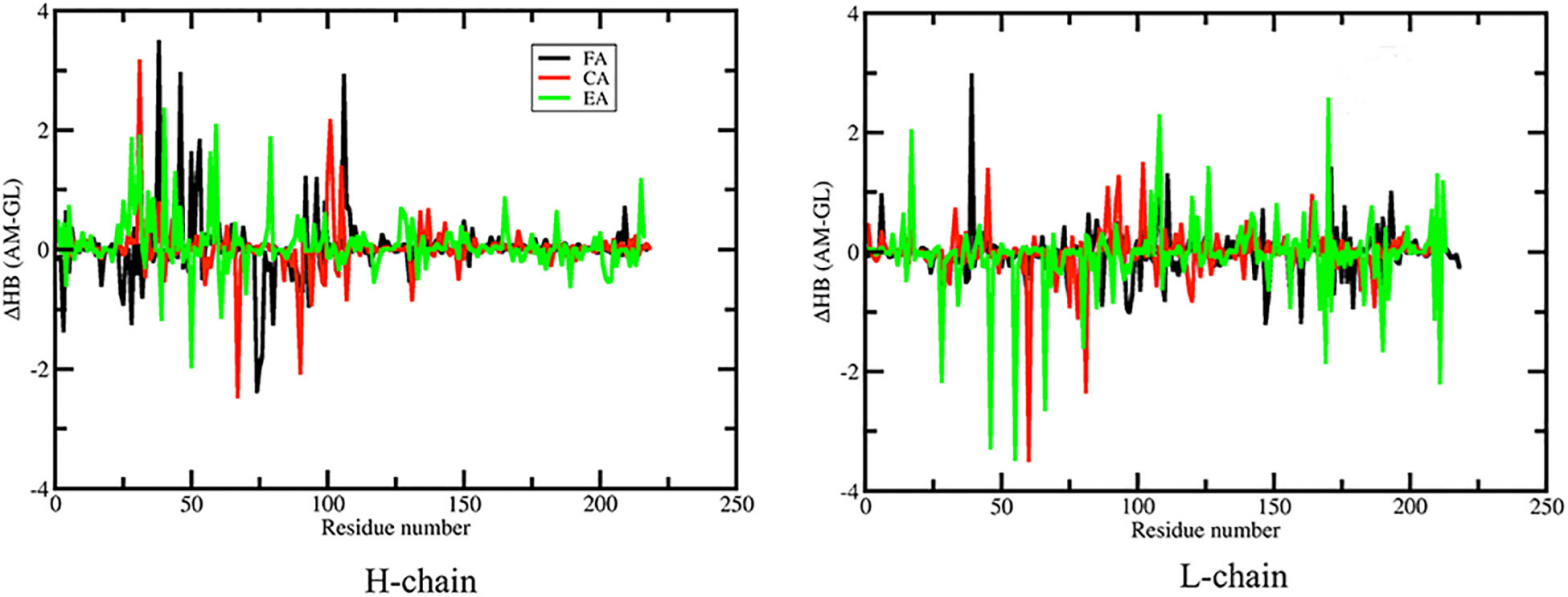
Differences in H-bond between GL and AM antibodies (A for H chain and B for L chain). The total H-bond counts (donor and acceptor) are averaged across the MD simulation for each residue. Positive values indicate AM antibodies have more hydrogen bonds than the GL antibodies and vice versa. FA, CA and EA represent anti-fluorescein, Anti-CD3 and esterase catalytic antibodies, respectively.

Fig 6 plots the difference of H-bonds for each residue between the GL and AM antibodies along the antibody sequences. Positive values represent more H-bonds formed in the AM antibodies, and negative values indicate more H-bonds in the GL antibodies. In the H chain, 27 residues gain at least one H-bond by somatic mutations and seven of them increase by two; simultaneously, 14 residues lose at least one H-bond, and three lose at least two H-bonds in the AM forms. Changes in the L chain are skewed in the opposite direction—20 residues gain at least one H-bond by somatic mutations (four residues gain two), while 21 residue lose H-bonds (7 of which decrease by two or more). This indicates that the somatic mutations significantly enhance the HBN in the H chain, but slightly weaken it in the L chain, which parallels the overall rigidity/flexibility changes.

To further characterize the relationship between the HBN and flexibility, we compare the Z-score versus the difference in H-bond propensity for each residue (S5 Fig). An important feature that cannot be over emphasized is that local H-bond propensity along the backbone does not correlate well to backbone flexibility. In particular, residues with significant rigidity/flexibility typically do not possess significant H-bond propensity changes due to the long-range nature of network rigidity. That is, rigidity changes from H-bond differences propagate through the network to affect distal residues mainly because H-bonds crosslink constraint topology. This crosslinking property of H-bond constraints distinguishes their effect from torsion constraints that model the influence of residue packing. Nevertheless, as shown in the figure, a local increase or decrease of two or more H-bonds at a specific residue location is a statistical indicator for a concomitant increase in rigidity or flexibility at that location. However, this statistical bias holds only for extreme outliers since most propensity changes are well within a two H-bond variation, and even for these outliers, exceptions remain. This result shows that the complexity of rigidity/flexibility changes is directly linked to the details of the HBN (or the entire constraint network) as a whole, rather than a local backbone characterization of the HBN. Some detailed cases are now considered and discussed.

**Fig 7:**
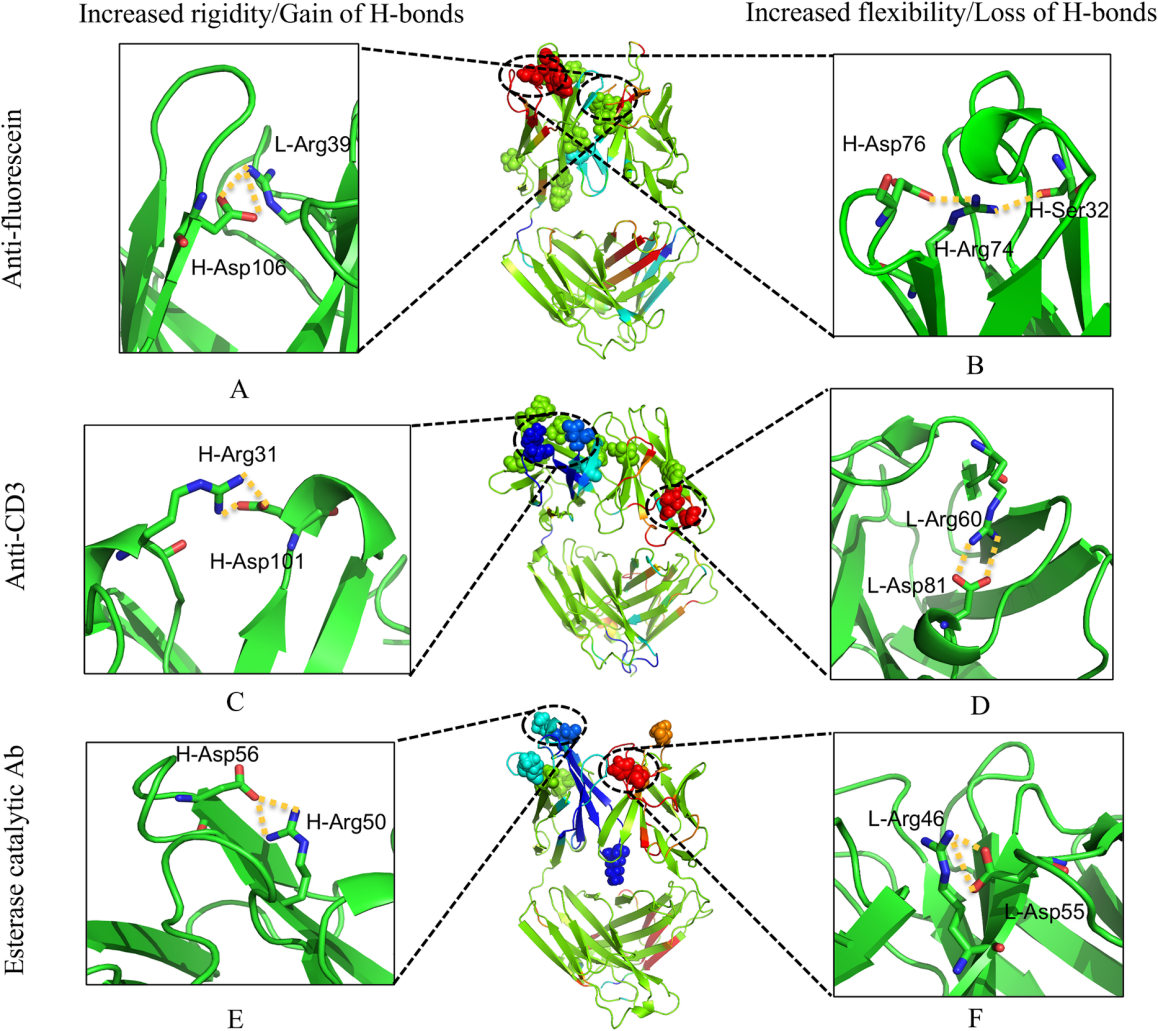
Relating flexibility differences to changes in the H-bond network. (A) and (B) are differences of hydrogen bonds for anti-fluorescein antibody, respectively. (C) and (D) for anti-CD3 antibody. (E) and (F) for esterase catalytic antibody. Regions that exhibit significant increased rigidity/flexibility are affected by gain/loss local H-bonds. Large increases in rigidity due to gain of H-bonds are displayed in the left panel while large increases in flexibility due to loss of H-bonds are in the right panel. The middle panel shows the same as in Fig 5. Hydrogen bonds are highlighted in yellow dashed lines.

Fig 7 demonstrates how mutations lead to significantly increased rigidity/flexibility by forming/breaking H-bonds. In FA, mutation VL H39R forms two new H-bonds to the VH Asp106 leading to increased rigidity within CDRs H3 (Fig 7A). In CA, the VH S31R and VH Y101D mutations form two new H-bonds leading to increased rigidity within CDRs H2 and H3, respectively (Fig 7C). Finally, in EA, mutation VH N56D forms two new H-bonds to the VH Arg50 leading to increased rigidity within CDRs H2 (Fig 7E). Therefore, in two of the three cases, the increased rigidity of CDR-H3 is mainly due to the local strengthening of the HBN. These local changes cause the whole loop to become rigid, meaning adjacent residues also become more rigid. This result demonstrates that the increased rigidity of CDR H3 could be obtained by introducing mutations directly forming H-bonds with the residues in this loop, which is in agreement with the multi-constraint design study for a set of antibodies that suggests proposed amino acid mutations along the CDR H3 loops for increasing the rigidity of the CDR H3 loop in the bound conformation to reduce its mobility [58]. By contrast, the increased rigidity of H3 in EA is not directly caused by an identified set of new H-bonds that form locally. Rather, increased rigidity in H3 is a consequence of a multitude of small but well distributed changes throughout the protein that supports the propagation of rigidity to H3.

Note that flexibility within L2 increases significantly in all three cases. In EA, the VL D55H mutation leads to the loss of two strong H-bonds between VL Asp55 and VL Arg46 (Fig 7F). Conversely, in FA and CA there are no significant local HBN differences. Increased flexibility upon affinity maturation is also observed in non-CDR loops. For example, increased flexibility in a pair of CA loops is associated with the loss of H-bonds between VL R60H and VL Asp81 (Fig 7D). Similarly, a non-CDR loop in the H chain (residue 74–79) of FA becomes more flexible due to the VH S32Ymutation within H1 (Fig 7B).

In the process of pinpointing specific H-bond differences that are responsible for the observed changes in flexibility, it is worth noting that Arg-to-Asp salt bridges do play an important role. In some cases, somatic mutations introduce both donor and acceptor (e.g. the VH S31R and VH Y101D pair of mutations in CA) or just one (e.g., the VL H39R in FA interacts with an Asp present in the GL antibody). Surprisingly, there are even examples of changes in Arg-Asp salt bridges occurring at positions that are not altered by affinity maturation, indicating that global changes in the network from the mutations occurring elsewhere lead to these new favorable interactions. For example, the salt bridge between VH Arg74 and VH Asp76 of FA is lost due to an aromatic mutation introduced within H1 that drastically affects the carboxylate side chain position of Asp76. Interestingly, it has been previously demonstrated that Arg-Asp salt bridges are the most frequent type occurring within antibody-antigen interfaces [59].

### Comparison of Mobility and Flexibility

**Fig 8:**
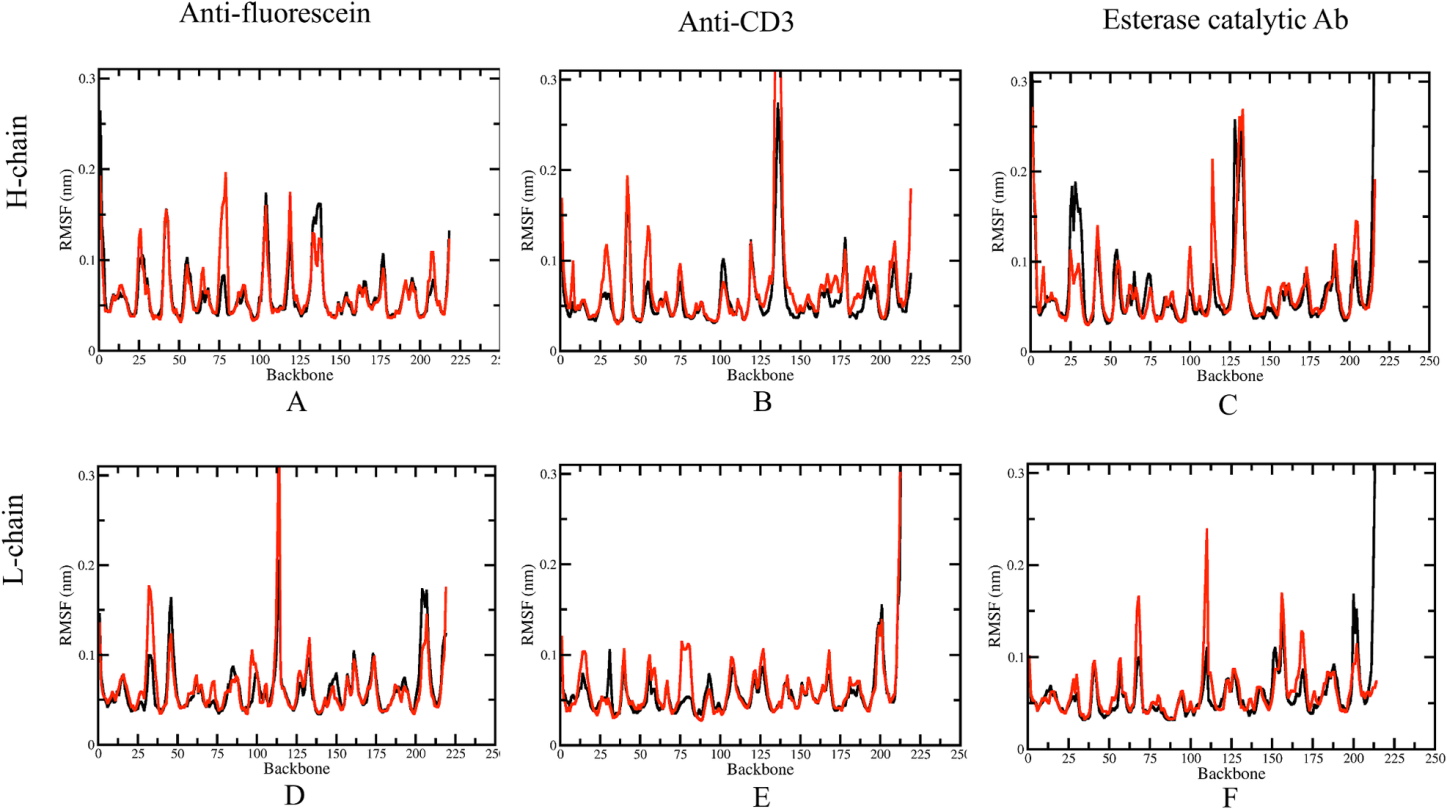
Conformational fluctuations. Residue root mean square fluctuations (RMSF) are provided for the six molecular dynamics trajectories (black indicates GL, whereas red indicates AM).

Keeping in mind that rigidity and flexibility fundamentally characterize different aspects of protein dynamics, Fig 8 shows the RMSFs of the three GL/AM antibodies. In FA and CA, the RMSFs of the GL CDR-H3 loops are both slightly higher than those of AM CDR-H3, revealing that the increased rigidity therein is accompanied by a decrease in motion. However, the corresponding RMSFs in EA actually increase upon maturation, meaning that rigid-body motions are present. S6 Fig shows the comparison of changes in flexibility and mobility by plotting the ΔRMSFs against ZSCORE. It is observed that decreased mobility can be consistent with decreased flexibility. This is especially true for the H chain of CA. A few residues with ΔRMSFs < -1 Å in the AM form possess ZSCORE < -2.3. However, in general, there is no significant correlation between the changes of mobility and flexibility, underscoring the differences between the two views of protein dynamics.

### Changes in Mechanical Couplings

Cooperativity correlation (CC) plots characterize mechanical couplings between residue pairs, providing a snapshot of allostery. It is worth pointing out that a particular rigid cluster can itself be very mobile as a rigid body, indicating the motion of all residues therein are highly correlated through the rigid body movement. When a pair of residues is flexibly correlated, random thermal motions of one residue is readily channeled into pathways dictated by how flexibility propagates through the protein to other residues, and vice versa. The rigidity network analysis highlights pathways defined by the native state ensemble of constraint topologies, but the mobility of atoms is not determined. Note, however, that molecular contacts can decrease mobility within flexible regions. As an analogy, a rigidity analysis would characterize the wiggling of fingers on a single hand as partly correlated, whereas the finger motions from two separate hands are uncorrelated. However, if the hands are clasped, the mobility of all fingers is greatly diminished due to being packed in an interlaced fashion. Thus, the CC-plot identifies channels of communication that are intrinsic to the skeletal structure of the protein, but the amplitude of motions that run through these channels is not quantified. In other words, thermodynamics and mechanics are quantified in QSFR, not kinetic properties.

The CC plots of the three antibody pairs are provided in S7 Fig, revealing that different domains are often flexibly correlated and the CDRs within each domain can be highly correlated as well. These correlations are expected to be important for function. In addition, except for the GL form of EA, the VH domain is primarily composed of one large rigid cluster, punctuated by several flexible loops. Conversely, the VL domain is significantly more co-flexible throughout the dataset. The CH domains are similar to the VH domains, but slightly more co-rigid. Across the dataset, the most co-rigid domains are the FA and CA CL domains.

**Fig 9:**
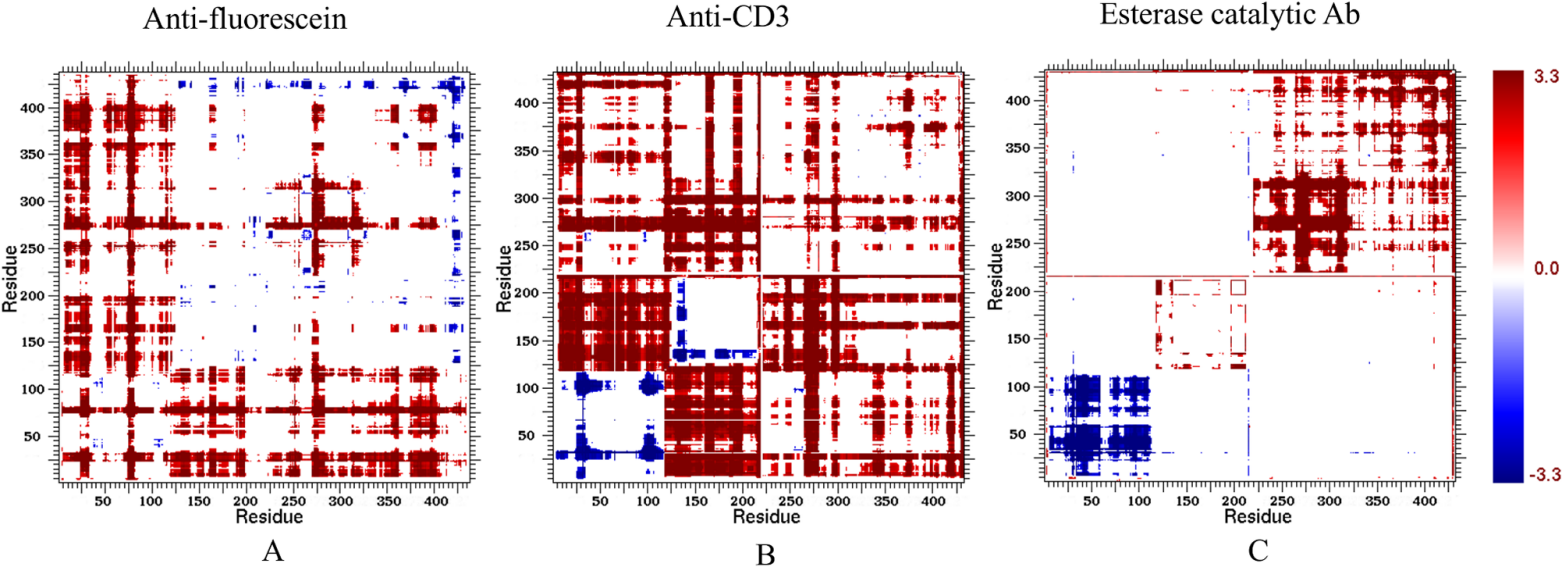
Changes in molecular cooperativity. Cooperativity correlation plots reveal intramolecular couplings within structure. That is, blue corresponds to residue pair correlated rigidity, whereas red correspond to correlated flexibility. White indicates no mechanical coupling between a pair of residues irrespective if the residues are flexible or rigid. For each case, the presented values represent the appropriate weighted average values over each set of ten representative structures sampled from the molecular dynamics trajectory.

Changes within CC highlight the sensitivity of rigidity properties to mutation, which is consistent with a number of our prior works [20, 21, 60]. Fig 9 plots ΔCC values represented by Z-scores per pixel for each of the antibody structures. Blue coloring indicates residue pairs that are more likely to be rigidly correlated, whereas red indicates residue pairs more likely to be flexibly correlated. Somatic mutations considerably increase rigid correlations between the CDRs of the VH domain such as the CDR-H1 and H3 in CA and all the three H-chain CDRs in EA. Meanwhile flexible correlations between different domains are enhanced. This result is consistent with a previous covariance analysis of molecular dynamic trajectories demonstrating that antibody motions in both CDRs and framework regions are correlated and that this correlation is stronger in the AM antibody [16]. Our results provide additional evidence that the correlations are enhanced during maturation, which makes sense due to the belief that these couplings are related to antigen specificity [61]. Note that most of the increases in backbone rigidity occur at locations where increased rigidly correlations also occur, indicating that the observed H-chain CDR rigidity increases are coupled. This leads to a cooperative mechanism that results in increased antigen specificity and affinity.

### Relating the Observed Changes to Antibody Evolution and Engineering

Starting from the GL antibody, the maturation process accumulates multiple mutations by repeated affinity maturation triggered by a specific antigen. GL antibodies are often polyspecific [6, 62–64], which is likely due to increased conformational flexibility. For example, comparisons bound and unbound GL antibodies show much greater conformational changes compared to the conformational changes that occurs within the corresponding AM pair [3]. Keeping with this, our results demonstrate that conformational flexibility is an intrinsic property of GL—specified CDR H3 sequences, and significant conformational change within the antibody-binding site especially restraining the H3 loop is the principal consequence of affinity maturation in the three considered examples. Antibody maturation typically accumulates multiple mutations (10–20) in the course of the conventional immune response by iterative mutation and selection triggered by a specific antigen. As expected, the large sets of GL AM mutants have greater collective responses compared to the relatively small number of mutations characterized in our previous study of the anti-lymphotoxin-**β** receptor antibody variants [26]. That is, successive cycles of mutations during the maturation process are needed to substantially alter flexibility characteristics of the Fab. Note that many somatic mutations across the dataset are located in the unstructured loops, which are more flexible than secondary structures (Fig 4). This suggests that the preferred introduction of mutations in GL loops during maturation is a primary driving force to the observed difference.

Interestingly, the observed changes across our dataset seldom significantly alter global flexibility properties within the antibody Fab and the antibody-binding site (Table 2 and Fig 5). For example, while VH is rigidified, the VL domain typically becomes more flexible. This near “zero-sum game” implies that the change of conformational diversity of antibody evolution again follows Le Châtelier’s principle [26]. That is, counteracting changes in rigidity and flexibility will occur at remote sites to globally restore the balance between rigidity and flexibility within protein structures [65]. Strikingly, our results show that both the co-rigid and co-flexible couplings between residues are enhanced during evolution, which suggest higher specificity require tighter collaboration between different structural components. This likely explains, at least in part, why multiple mutations are required for affinity maturation.

Computation-guided affinity maturation is an appealing approach toward antibody engineering. Currently, most efforts focus on improving the association between receptor and substrate by optimizing their interactions using force-field-based energy functions [66–70]. However, the current success rate is rather modest because of the inaccuracy of force fields and complexity of interaction network. The mechanism of evolution-mediated conformational changes provides common features of affinity-matured antibodies that could be tracked to guide the design of high affinity antibodies by introducing local and distal mutations. One can imagine that simply introducing local mutations that rigidify the desired CDR loops to fix the optimal binding site conformations could increase the binding affinity to an antigen. This is partially true because the local neighborhood of a mutation will accommodate the new residue respecting local geometrical constraints and network constraints imposed by the protein. However, the final effect on rigidity and flexibility is a mix of both strengthening and weakening effects that occur over both short and long distances. Given that the molecular details that involve multiple mutations during evolution are complicated, a rapid high throughput computational method that does not rely on local propensity properties is required. The mDCM has provided insight into the process, and may provide a useful tool to assess the effects of mutations within antibody design algorithms. In ongoing work, we are characterizing how mutations affect the allosteric response to surface interactions within antibody fragments. These characterizations reveal a similarly diverse set of antibody-substrate interactions, meaning that antibody maturation likely also has a significant effect on intramolecular couplings. Future work will determine if this is the case.

## Supporting Information

S1 Table Assignment of germline gene for the three antibodies. (DOCX)

S2 Table Experimental binding affinities for the three antibody pairs. (DOCX)

S3 Table Summary of somatic mutations across the dataset. (DOCX)

S4 Table mDCM parameters for the three GL-AM pairs. (DOCX)

S5 Table Statistic of changes of amino acid propensity during affinity maturation in the dataset. (DOCX)

S1 Fig Summary of the mutation types. Counts of the type of amino acid mutations before (blue) and after (red) affinity maturation across the dataset.
(TIF)

S2 Fig Conformational fluctuations. Molecular dynamics RMSDs are plotted for each germline (GL) and affinity mature (AM) antibody systems along the 100 ns trajectories. In each case, the RMSDs are provided for the full Fab fragment and also for each constituent domain. (A) GL anti-fluorescein antibody, (B) AM anti-fluorescein antibody, (C) GL anti-CD3 antibody (D) AM anti-CD3 antibody, (E) GL esterase catalytic antibody, and (F) AM mature esterase catalytic antibody.
(TIF)

S3 Fig Changes in backbone flexibility. Changes in backbone flexibility are indicated by z-scores using Eq. (3) from above. Positive values correspond to increased flexibility within the mutant, whereas negative values correspond to increased rigidity. Values within the range of ±2.33 are considered to have no change; values of ± (2.33–3.33) are considered to have moderate changes; and values beyond ±3.33 define large changes. The z-score representation of differences in backbone flexibility quantifies the significance of the observed changes that include both local and non-local changes in rigidity or flexibility.
(TIF)

S4 Fig H-bond network visualization. The H-bond networks across the dataset are shown, where white nodes denote H-bond donor and acceptor atoms, and colored edges represent H-bond occupancy across the molecular dynamics simulation trajectory. Black corresponds to H-bonds present greater than 90% of the simulation; blue corresponds to 70–90%; and green corresponds to 50–70%. Because we are primarily interested in stronger H-bonds, those present less than 50% of the time are not shown.
(TIF)

S5 Fig Comparing changes in flexibility as a z-score to changes in H-bond propensities for each residue. Points outside the rectangles as dashed red lines represent residues with significant differences of H-bonds (count of H-bonds < = -1 or > = 1) and/or flexibility (|Z-score| < = -2.33 or > = 2.33).
(TIF)

S6 Fig Changes in mechanical flexibility (z-score) are compared to changes in the dynamical RMSF for each residue across the dataset. (TIF)

S7 Fig Relating changes in the H-bond network to changes in flexibility. Cooperativity correlation difference plots highlight differences in pairwise mechanical couplings between the wild type and each mutant. Red indicates increased correlated flexibility within the mutant structure, whereas blue indicates increased correlated rigidity. White indicates no change. Notice in most mutants (i.e., triple mutant), changes in cooperativity correlation occur throughout the Fv structure, whereas they are primarily isolated to the VH domain in the quadruple mutant.
(TIF)
