# Supplementary material for: Rigidity Emerges during Antibody Evolution in Three Distinct Antibody Systems: Evidence from QSFR Analysis of Fab Fragments
Source: PLoS Comput Biol. 2015 Jul 1;11(7):e1004327. doi: 10.1371/journal.pcbi.1004327 (PMC4489365; doi:10.1371/journal.pcbi.1004327)
Supplement: S1 Table — (DOCX) [file pcbi.1004327.s001.docx]

S1 Table. Assignment of germline gene for the three antibodies.

| Antibody Fab | Heavy chain^a^ | | | Light chain^a^ | |
| --- | --- | --- | --- | --- | --- |
|  | V | D^b^ | J | V | J |
| Anti-Fluorescein | IGHV3 | N/A | IGHJ6 | IGKV2 | IGKJ4 |
| Anti-CD3 | IGHV1 | N/A | IGHJ6 | IGKV3 | IGKJ2 |
| Esterolytic catalytic Ab | IGHV1 | N/A | IGHJ6 | IGKV1 | IGKJ4 |

a. The germline genes were assigned by bioinformatics analysis of the closet human germline sequence using the following online web http://www.bioinf.org.uk/abysis/index.html and antibody genes were named based on nomenclature of IMGT (http://www.imgt.org/).

b. It is difficult to assign the germline D gene due to its diversity.
