# Supplementary material for: Rigidity Emerges during Antibody Evolution in Three Distinct Antibody Systems: Evidence from QSFR Analysis of Fab Fragments
Source: PLoS Comput Biol. 2015 Jul 1;11(7):e1004327. doi: 10.1371/journal.pcbi.1004327 (PMC4489365; doi:10.1371/journal.pcbi.1004327)
Supplement: S2 Table — (DOCX) [file pcbi.1004327.s002.docx]

S2 Table. Experimental binding affinities for the three antibody pairs.

| Antibody Fab | GL/AM | K_d_ (μM) |
| --- | --- | --- |
| Anti-Fluorescein | GL | 7-150 [[2](#_ENREF_2),[3](#_ENREF_3)]^a^ |
|  | AM | 0.22 [[3](#_ENREF_3)] |
| Anti-CD3 | GL | No binding [[4](#_ENREF_4)] |
|  | AM | 0.64 [[5](#_ENREF_5)] |
| Esterolytic catalytic Ab | GL | 0.330 [[6](#_ENREF_6)] |
|  | AM | 0.045 [[6](#_ENREF_6)] |

a. The binding affinity of anti-fluorescein antibody with the germline sequences in both heavy and light chains are not available. Here a range of K_d_ values corresponding to FA antibodies with a single mutation away from GL is provided.
