# Supplementary material for: Rigidity Emerges during Antibody Evolution in Three Distinct Antibody Systems: Evidence from QSFR Analysis of Fab Fragments
Source: PLoS Comput Biol. 2015 Jul 1;11(7):e1004327. doi: 10.1371/journal.pcbi.1004327 (PMC4489365; doi:10.1371/journal.pcbi.1004327)
Supplement: S3 Table — (DOCX) [file pcbi.1004327.s003.docx]

S3 Table. Summary of somatic mutations across the dataset.

| Antibody Fab | chain | GL---->AM mutations |
| --- | --- | --- |
| Anti-fluorescein | H | S17P, T30S, N31D,S32Y,F37V,C38R, K52R, K53N, A84V, T87M |
|  | L | H39R, L51V |
| Anti-CD3 | H | S31R, S55R, K59N, A72T, Y101D, F106L |
|  | L | H34N, R61H, P96F |
| Esterolytic catalytic Ab | H | E42K, G55V, N56D, G65D, N76K, A78T |
|  | L | S30N, S34G, D55H |
