# Supplementary material for: Rigidity Emerges during Antibody Evolution in Three Distinct Antibody Systems: Evidence from QSFR Analysis of Fab Fragments
Source: PLoS Comput Biol. 2015 Jul 1;11(7):e1004327. doi: 10.1371/journal.pcbi.1004327 (PMC4489365; doi:10.1371/journal.pcbi.1004327)
Supplement: S4 Table — (DOCX) [file pcbi.1004327.s004.docx]

S4 Table. mDCM Parameters for the three GL-AM pairs.

| Antibody Fab | GL/AM | δ_nat_ | u_sol_ | ν_nat_ |
| --- | --- | --- | --- | --- |
| Anti-fluorescein | GL | 1.94 | -2.24 | -0.36 |
|  | AM | 1.94 | -2.24 | -0.36 |
| Anti-CD3 | GL | 1.94 | -2.40 | -0.74 |
|  | AM | 1.94 | -2.37 | -0.68 |
| Esterolytic catalytic Ab | GL | 1.94 | -2.24 | -0.36 |
|  | AM | 1.94 | -2.24 | -0.36 |
