# Supplementary material for: Rigidity Emerges during Antibody Evolution in Three Distinct Antibody Systems: Evidence from QSFR Analysis of Fab Fragments
Source: PLoS Comput Biol. 2015 Jul 1;11(7):e1004327. doi: 10.1371/journal.pcbi.1004327 (PMC4489365; doi:10.1371/journal.pcbi.1004327)
Supplement: S5 Table — (DOCX) [file pcbi.1004327.s005.docx]

S5 Table. Statistic of changes of amino acid propensity during affinity maturation in the dataset.

| Stage^a^ | Aliphatic | Aromatic | Polar | Charged |
| --- | --- | --- | --- | --- |
| Before | 7 | 3 | 15 | 7 |
| After | 9 | 2 | 7 | 14 |

^a^ Before or after the design
