# Supplementary figures and images for: Rigidity Emerges during Antibody Evolution in Three Distinct Antibody Systems: Evidence from QSFR Analysis of Fab Fragments
Source: PLoS Comput Biol. 2015 Jul 1;11(7):e1004327. doi: 10.1371/journal.pcbi.1004327 (PMC4489365; doi:10.1371/journal.pcbi.1004327)

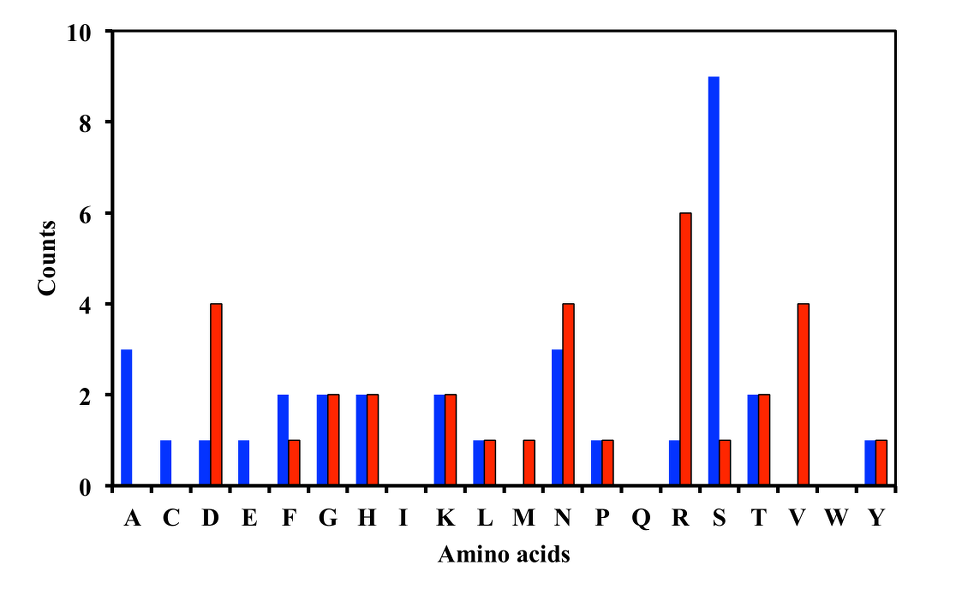

Supplement: S1 Fig — Counts of the type of amino acid mutations before (blue) and after (red) affinity maturation across the dataset. (TIF) [file pcbi.1004327.s006.tif]

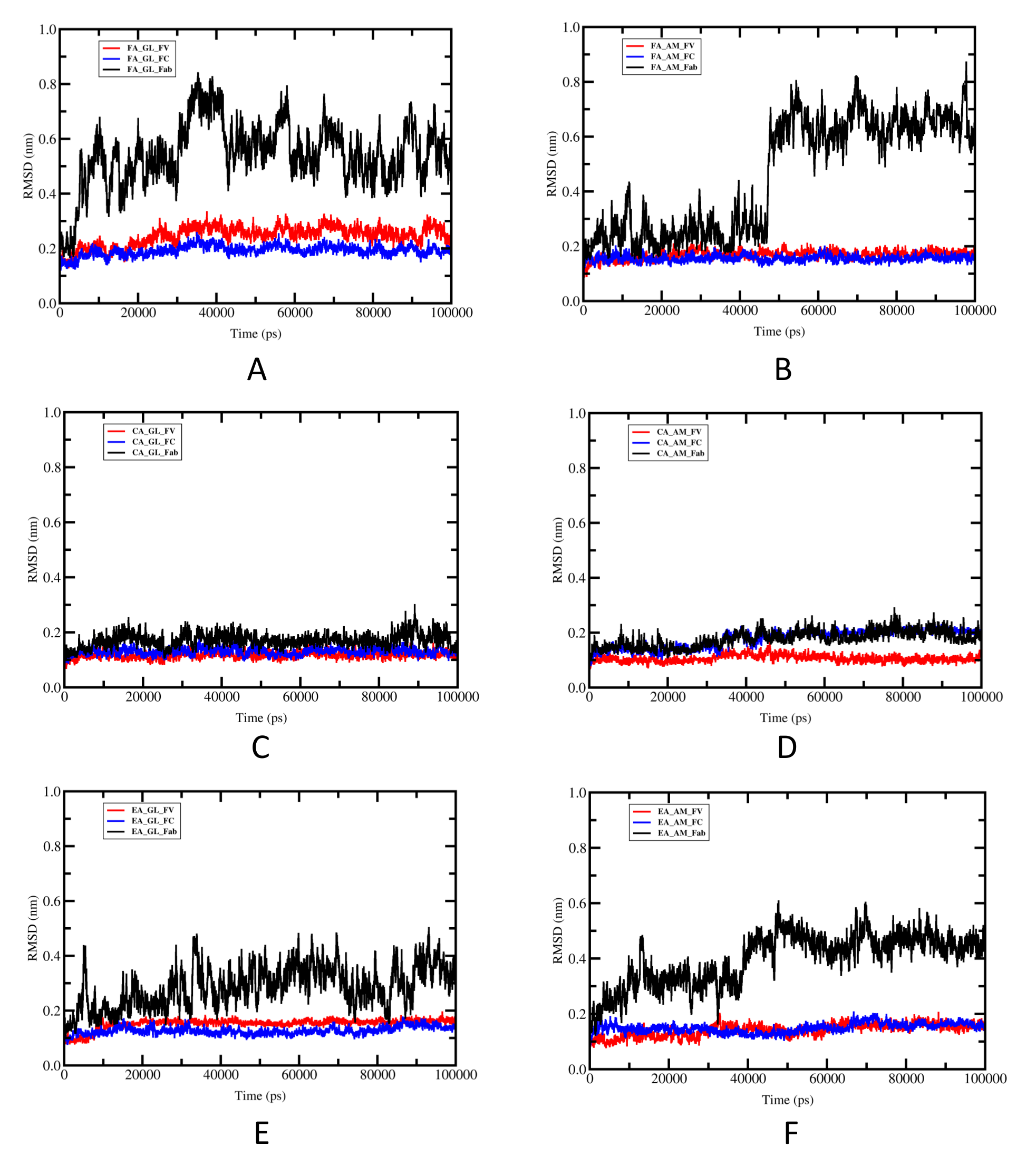

Supplement: S2 Fig — Molecular dynamics RMSDs are plotted for each germline (GL) and affinity mature (AM) antibody systems along the 100 ns trajectories. In each case, the RMSDs are provided for the full Fab fragment and also for each constituent domain. (A) GL anti-fluorescein antibody, (B) AM anti-fluorescein antibody, (C) GL anti-CD3 antibody (D) AM anti-CD3 antibody, (E) GL esterase catalytic antibody, and (F) AM mature esterase catalytic antibody. (TIF) [file pcbi.1004327.s007.tif]

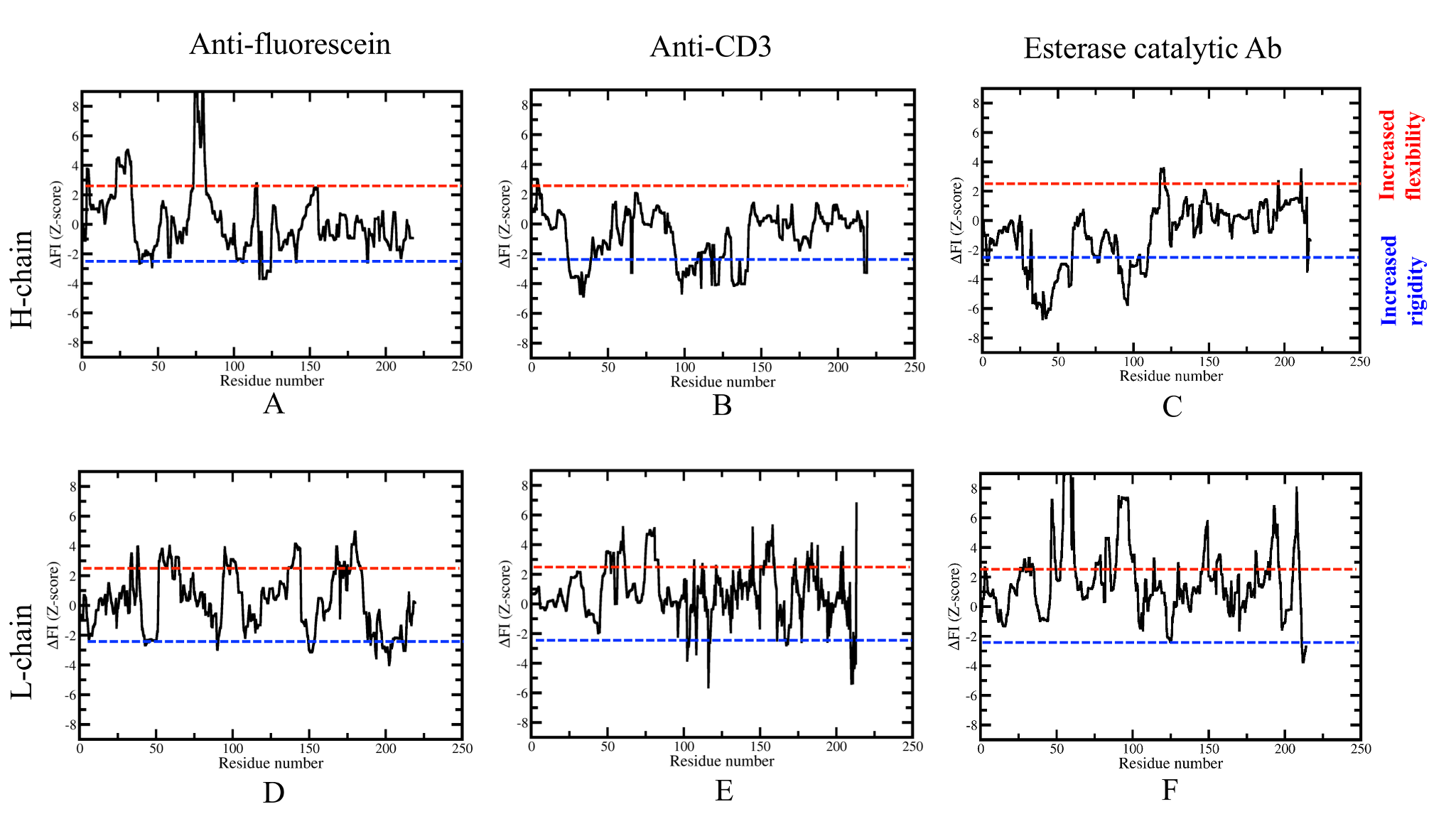

Supplement: S3 Fig — Changes in backbone flexibility are indicated by z-scores using Eq. (3) from above. Positive values correspond to increased flexibility within the mutant, whereas negative values correspond to increased rigidity. Values within the range of ±2.33 are considered to have no change; values of ± (2.33–3.33) are considered to have moderate changes; and values beyond ±3.33 define large changes. The z-score representation of differences in backbone flexibility quantifies the significance of the observed changes that include both local and non-local changes in rigidity or flexibility. (TIF) [file pcbi.1004327.s008.tif]

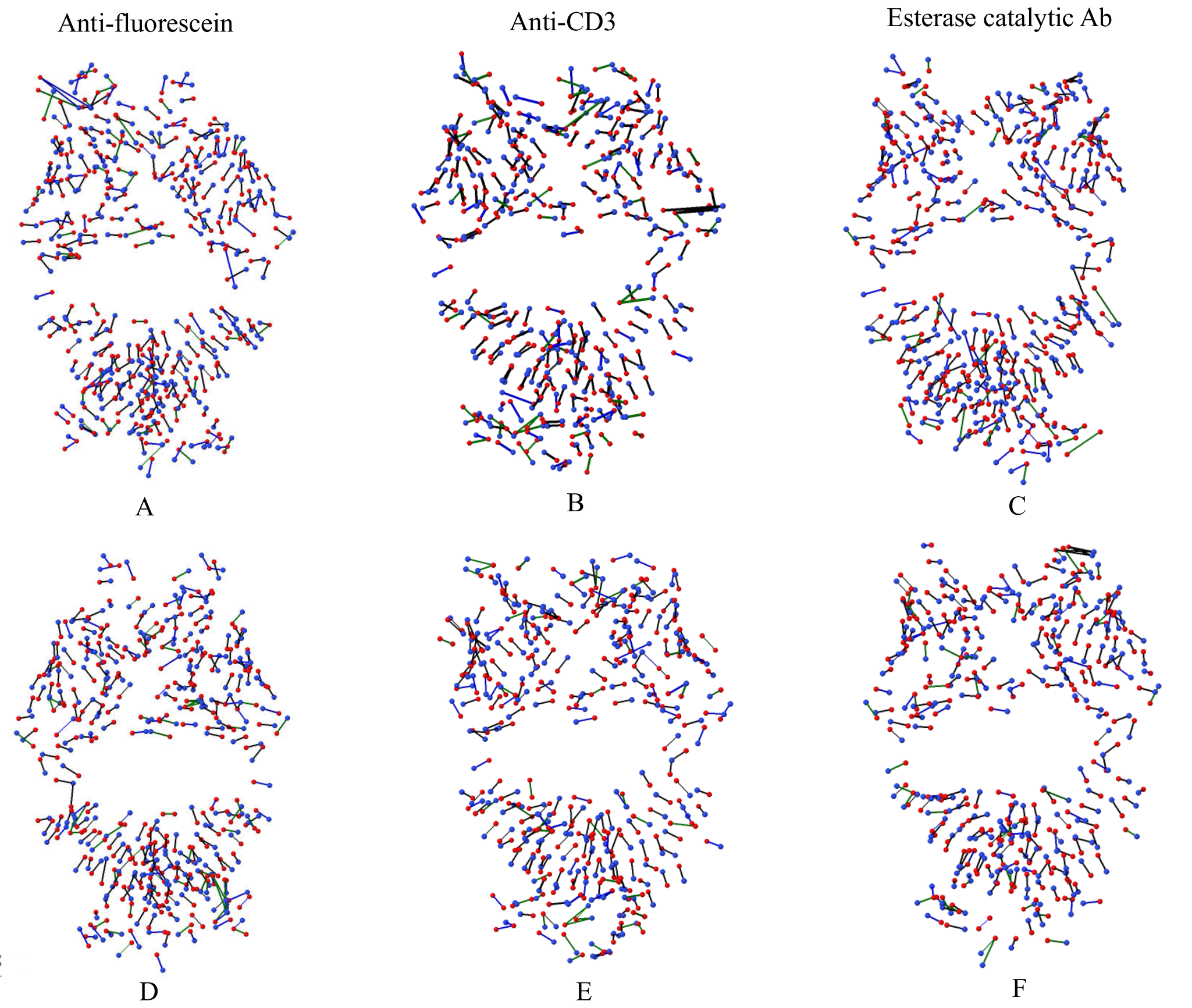

Supplement: S4 Fig — The H-bond networks across the dataset are shown, where white nodes denote H-bond donor and acceptor atoms, and colored edges represent H-bond occupancy across the molecular dynamics simulation trajectory. Black corresponds to H-bonds present greater than 90% of the simulation; blue corresponds to 70–90%; and green corresponds to 50–70%. Because we are primarily interested in stronger H-bonds, those present less than 50% of the time are not shown. (TIF) [file pcbi.1004327.s009.tif]

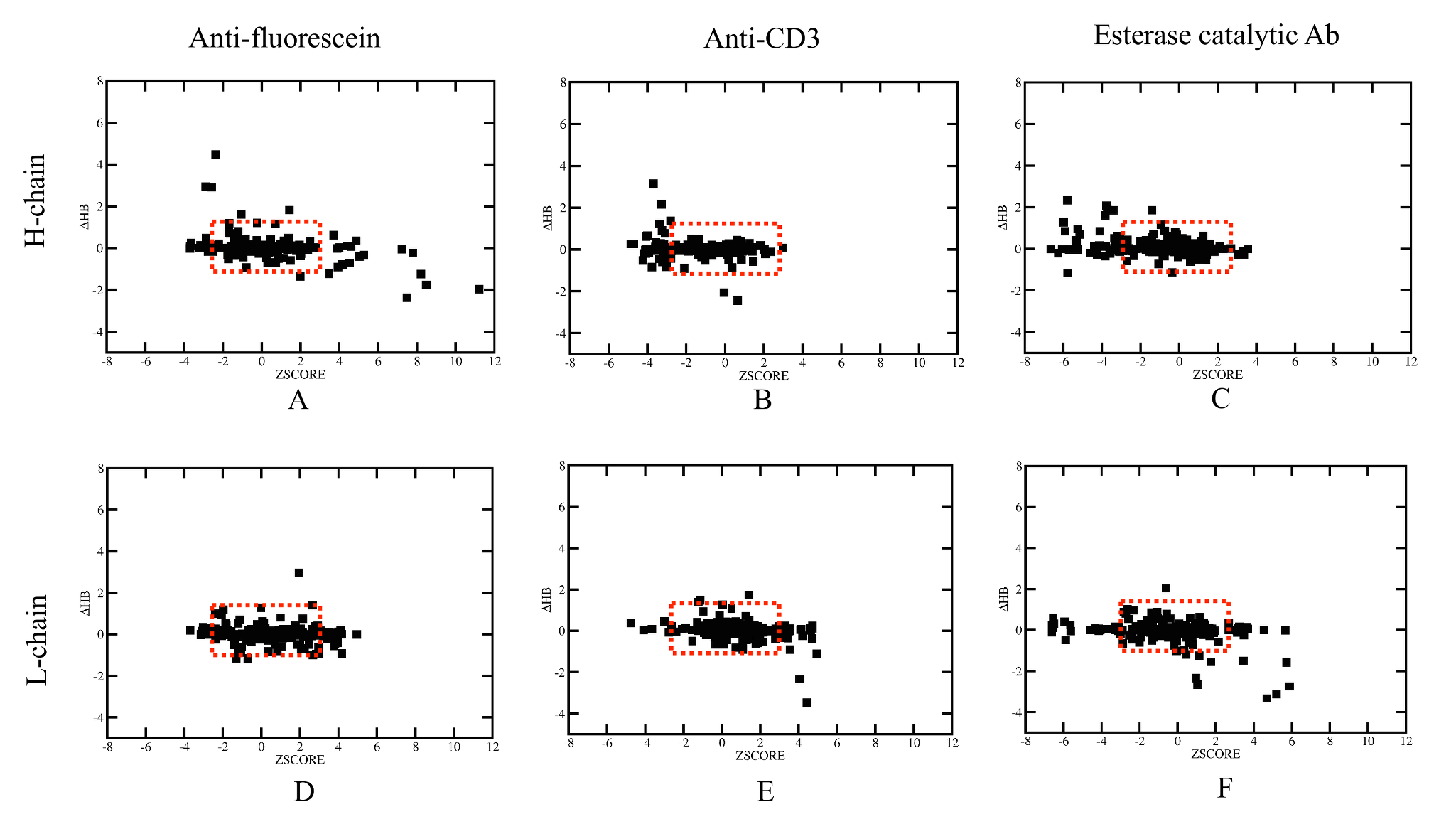

Supplement: S5 Fig — Points outside the rectangles as dashed red lines represent residues with significant differences of H-bonds (count of H-bonds < = -1 or > = 1) and/or flexibility (|Z-score| < = -2.33 or > = 2.33). (TIF) [file pcbi.1004327.s010.tif]

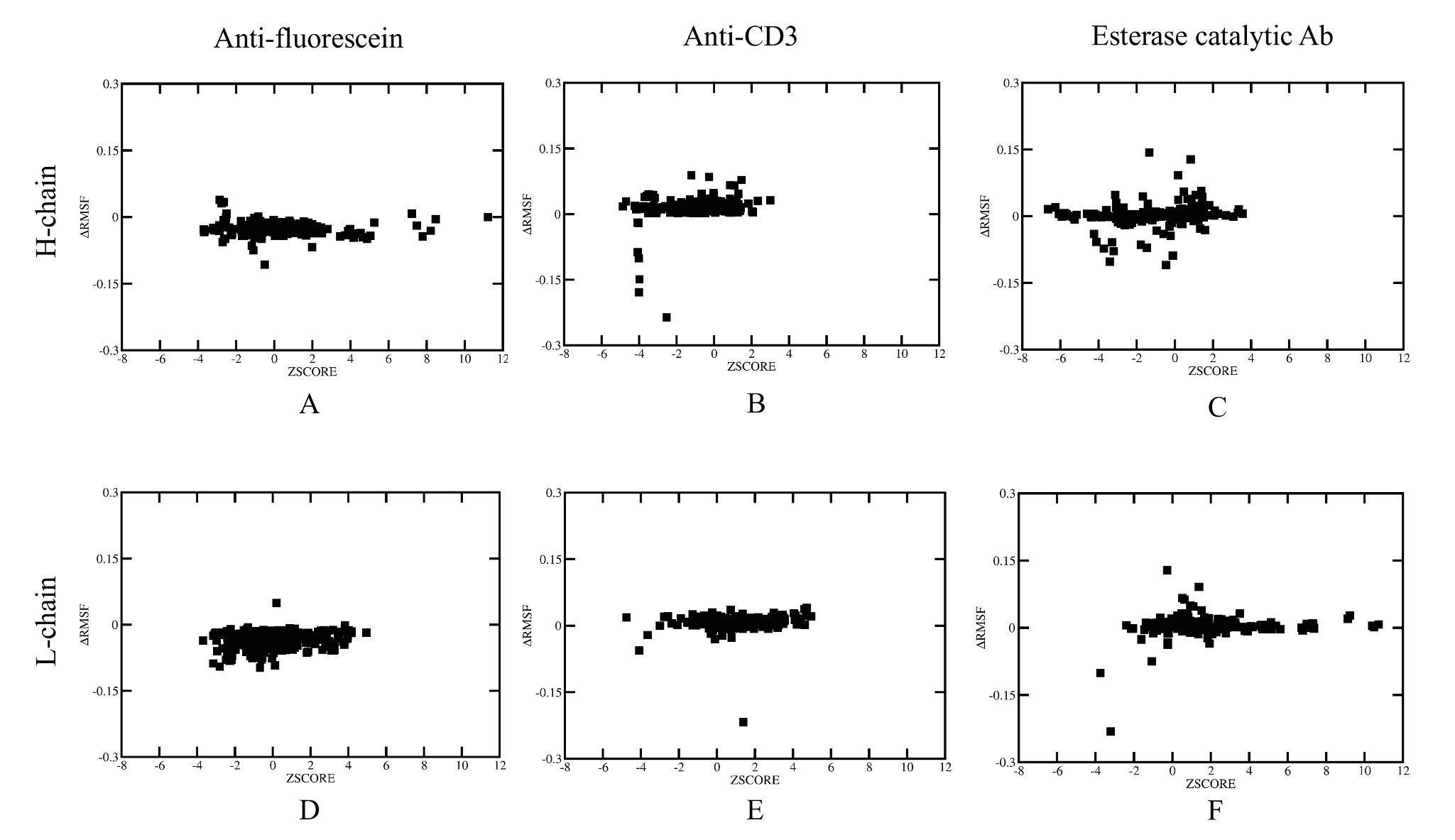

Supplement: S6 Fig — (TIF) [file pcbi.1004327.s011.tif]

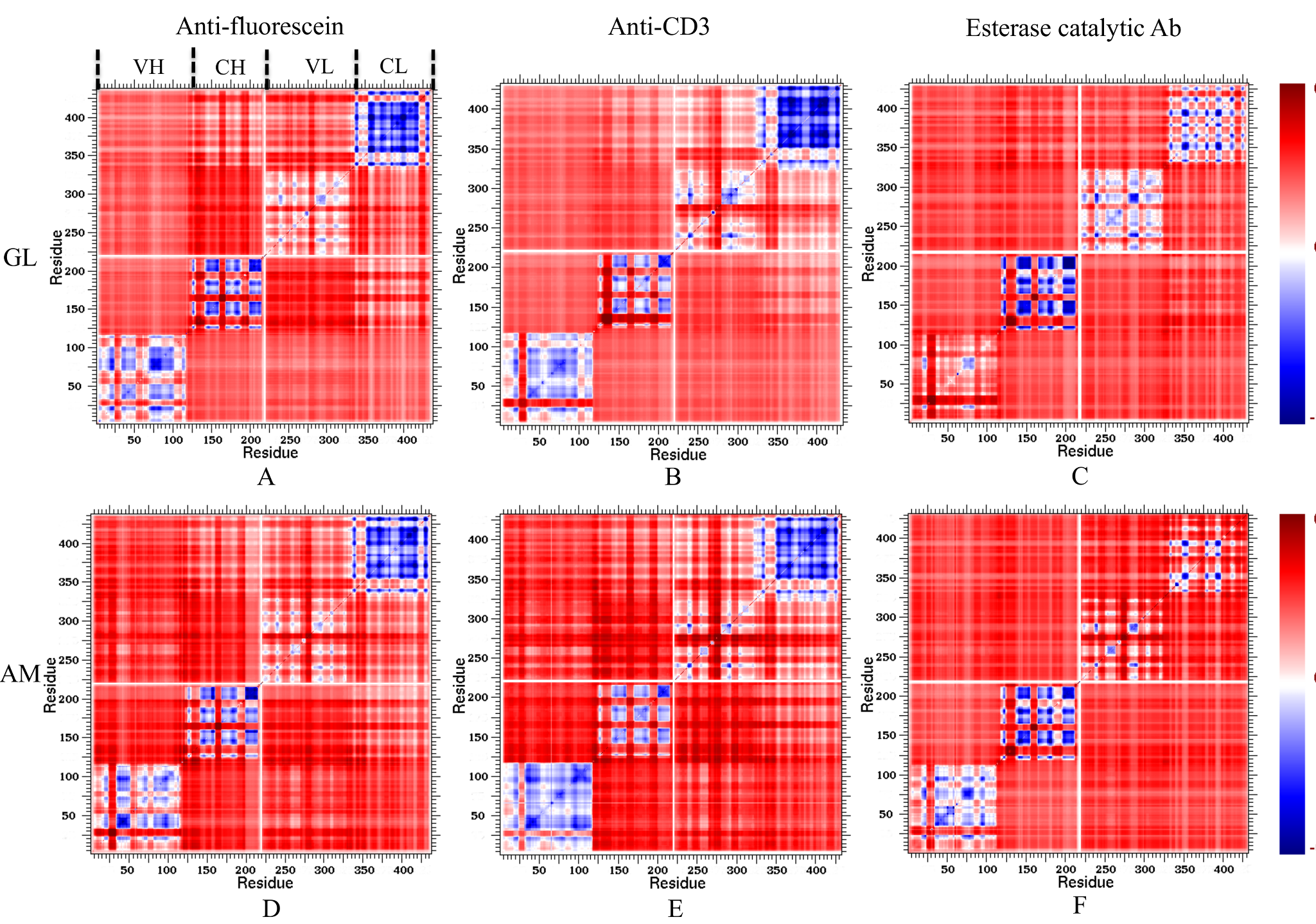

Supplement: S7 Fig — Cooperativity correlation difference plots highlight differences in pairwise mechanical couplings between the wild type and each mutant. Red indicates increased correlated flexibility within the mutant structure, whereas blue indicates increased correlated rigidity. White indicates no change. Notice in most mutants (i.e., triple mutant), changes in cooperativity correlation occur throughout the Fv structure, whereas they are primarily isolated to the VH domain in the quadruple mutant. (TIF) [file pcbi.1004327.s012.tif]
